# The AtRAD21.1 and AtRAD21.3 Arabidopsis cohesins play a synergistic role in somatic DNA double strand break damage repair

**DOI:** 10.1186/s12870-014-0353-9

**Published:** 2014-12-16

**Authors:** José A da Costa-Nunes, Cláudio Capitão, Jaroslav Kozak, Pedro Costa-Nunes, Gloria M Ducasa, Olga Pontes, Karel J Angelis

**Affiliations:** Instituto de Tecnologia Química e Biológica (ITQB), Universidade Nova de Lisboa (UNL), Av. República, Apartado 127, 2781-901 Oeiras, Portugal; Laboratório de Biotecnologia de Células Vegetais, ITQB, UNL, Av. República, Apartado 127, 2781-901 Oeiras, Portugal; Current address: Gregor Mendel Institute of Molecular Plant Biology, Austrian Academy of Sciences, Vienna Biocenter, 1030 Vienna, Austria; Molecular Farming Lab., Institute of Experimental Botany AS CR, Na Karlovce 1, 160 00 Praha 6, Czech Republic; Department of Biology, University of New Mexico, 235 Castetter Hall, MSC03 2020, 1 University of New Mexico, Albuquerque, NM 87131-0001 New Mexico USA; Current address: Nuclear Organization and Epigenetics Lab., Shanghai Center for Plant Stress Biology (PSC), No. 3888 Chenhua Road, Shanghai, 201602 P. R. China

**Keywords:** Arabidopsis, *AtRAD21.1*, *AtRAD21.3*, Cohesins, Comet assay, DNA damage, Gene expression

## Abstract

**Background:**

The RAD21 cohesin plays, besides its well-recognised role in chromatid cohesion, a role in DNA double strand break (dsb) repair. In *Arabidopsis* there are three *RAD21* paralog genes (*AtRAD21.1*, *AtRAD21.2* and *AtRAD21.3*), yet only *AtRAD21.1* has been shown to be required for DNA dsb damage repair. Further investigation of the role of cohesins in DNA dsb repair was carried out and is here reported.

**Results:**

We show for the first time that not only AtRAD21.1 but also AtRAD21.3 play a role in somatic DNA dsb repair. Comet data shows that the lack of either cohesins induces a similar high basal level of DNA dsb in the *nuclei* and a slower DNA dsb repair kinetics in both cohesin mutants. The observed *AtRAD21.3* transcriptional response to DNA dsb induction reinforces further the role of this cohesin in DNA dsb repair. The importance of AtRAD21.3 in DNA dsb damage repair, after exposure to DNA dsb damage inducing agents, is notorious and recognisably evident at the phenotypical level, particularly when the *AtRAD21.1* gene is also disrupted.

Data on the kinetics of DNA dsb damage repair and DNA damage sensitivity assays, of single and double *atrad21* mutants, as well as the transcription dynamics of the AtRAD21 cohesins over a period of 48 hours after the induction of DNA dsb damage is also shown.

**Conclusions:**

Our data demonstrates that both *Arabidopsis* cohesin (AtRAD21.1 and AtRAD21.3) play a role in somatic DNA dsb repair. Furthermore, the phenotypical data from the *atrad21.1 atrad21.3* double mutant indicates that these two cohesins function synergistically in DNA dsb repair. The implications of this data are discussed.

**Electronic supplementary material:**

The online version of this article (doi:10.1186/s12870-014-0353-9) contains supplementary material, which is available to authorized users.

## Background

RAD21 (also known as SCC1) [1,2], SMC1, SMC3 and SCC3 are the core subunits of a complex required for sister chromatid cohesion [3]. Sister chromatid cohesion in budding yeast is established during late G1 and S phase [4,5] and is abolished during the metaphase/anaphase transition, to allow the correct and timely mitotic sister chromatid segregation [6]. Sister chromatid cohesion is also established *de novo* during the G2/M diploid phases when DNA dsb are formed [5,7]. This *de novo* cohesion induced by DNA dsb occurs in budding yeast on a genome-wide scale [7,8]. In contrast, in human cells at the G2 phase, the RAD21 cohesin is recruited and targeted specifically to the vicinity of the DNA dsb *loci* [9,10]. It has been proposed that the *de novo* cohesion establishment tethers the DNA dsb damaged strand with its identical and intact sister chromatid counterpart to promote error-free DNA repair [7].

DNA dsb can be repaired via different DNA repair pathways such as the error-free homologous recombination (HR) pathway, which requires a homologous DNA strand template for repair, or via other alternative DNA dsb repair pathways that do not require a homologous template. The latter, such as the canonical non-homologous end-joining (C-NHEJ), the single strand annealing and the micro-homology end-joining DNA repair pathways are mostly error-prone [11,12]. In imbibed seeds, for example, DNA dsb can be repaired via different DNA dsb repair pathways. Accordingly, mutations that affect either HR or C-NHEJ have been reported to cause loss of viability, or developmental delay, in seedlings germinated from imbibed mutants seeds of *Arabidopsis thaliana* (henceforth Arabidopsis) and maize exposed to DNA dsb damage inducing agents [13-16].

Other than triggering *de novo* cohesion, DNA dsb damage also triggers changes in gene expression. Some of the *Arabidopsis* genes that code for proteins required at the early stages of HR repair of DNA dsb, such as *AtRAD51*, *AtBRCA1*, *AtRPA-related*, *AtGR1*/*COM1/CtIP* and *GMI1*, increase gene expression after DNA dsb induction [17-23]. Yet, not all Arabidopsis genes involved in HR, namely *AtRAD50* and *AtNBS1* (which are also involved in C-NHEJ), are transcriptionally responsive to DNA dsb damage [21,22,24,25]. DNA dsb damage also induces increase of the expression levels of the *AtWEE1*, *CycB1:1* and *AtRAD17*, genes that are involved in cell cycle arrest at G2 [21,26,27]. This DNA dsb induced G2 cell cycle arrest is detected mainly in meristems [21,22,28,29]. The observed increase of steady-state transcript levels, induced by DNA dsb, of the genes mentioned above and of *AtRAD21.1* is mediated by the ATM kinase [21,30].

Arabidopsis has three *RAD21* homologous genes; *AtRAD21.1/SYN2*, *AtRAD21.1.2/SYN3* and *AtRAD21.3/SYN4* [14,31]. *AtRAD21.1* transcripts are detected in low levels in most plant tissues [14,32], yet in the shoot apex and particularly in seeds (and more so in dry and imbibed seeds), higher levels of *AtRAD21.1* transcript can be found [33-35]. *AtRAD21.1* transcripts become more abundant upon DNA dsb induction, in an ATM dependent manner, after DNA dsb induction [14,20,21]. The detection of higher *AtRAD21.1* expression levels in seeds and the shoot apical apex is particularly interesting since these contain actively dividing meristem cells where maintenance of genomic integrity is crucial. Like *AtRAD21.1*, the *AtRAD21.2* gene is also expressed in different tissues at low levels [14,31]. Yet, and unlike *AtRAD21.1*, *AtRAD21.2* steady-state transcript levels have been shown not to increase in response to DNA dsb damage induction [14]. In contrast, the cohesin *AtRAD21.3* exhibits the highest steady-state transcript levels of all *AtRAD21* genes [14]. AtRAD21.3 has been shown to play a role in genome stability and to be associated with replication factors [36]. Indeed, the *atrad21.3* mutant experiences genomic instability (like *atrad21.1*) and chromatid alignment defects [37], yet, unlike the *atrad21.1* mutant, the *atrad21.3* single mutant has not been reported to be associated with DNA dsb damage repair nor to exhibit a DNA dsb damage hypersensitivity phenotype [14]. However, and unexpectedly, AtRAD21.3 is involved in DNA dsb damage repair.

Here, we report for the first time that AtRAD21.3, like AtRAD21.1, also plays a role in somatic DNA dsb repair. Both *atrad21.3* and *atrad21.1* single mutants have a higher basal level of DNA dsb, in comparison to wild-type Columbia-0 (Col). Additionally, the *atrad21.3* mutation also affects the kinetics of DNA dsb damage repair after the induction of DNA dsb. Furthermore, the combination of both mutations renders the imbibed seeds of the *atrad21.1 atrad21.3* double mutant more hypersensitive to DNA dsb induction than the *atrad21.1* and the *atrad21.3* single mutants.

We also show that the emergency-like *AtRAD21.1* gene expression response to DNA damage is triggered immediately and abruptly after the induction of DNA dsb.

## Results

### The *AtRAD21.1* complementation construct is sufficient to promote resistance to ionising radiation-induced damage in imbibed seeds

The *atrad21.1* mutation (salk_044851) renders Arabidopsis imbibed seeds hypersensitive to DNA dsb-inducing agents [14]. To establish that the described phenotype is caused by the *atrad21.1* mutation alone, and not derived from chromosomal rearrangement or the disruption of another gene not physically linked to the T-DNA insertion [38], *atrad21.1* mutant plants were transformed with the complementation construct.

To obtain the complementation construct, the genomic region comprising the *AtRAD21.1* gene and its 2,602 bp upstream sequence, was amplified as a single PCR product and cloned. Sequencing of the genomic complementation construct confirmed that the coding sequence in the construct is identical to the coding sequence of the *AtRAD21.1* wild-type allele. Sequencing also confirmed that the complementation construct *AtRAD21.1* gene sequence is cloned in frame with the epitope-tags *GFP*-6x*His* (from the pMDC107 vector).

**Figure 1:**
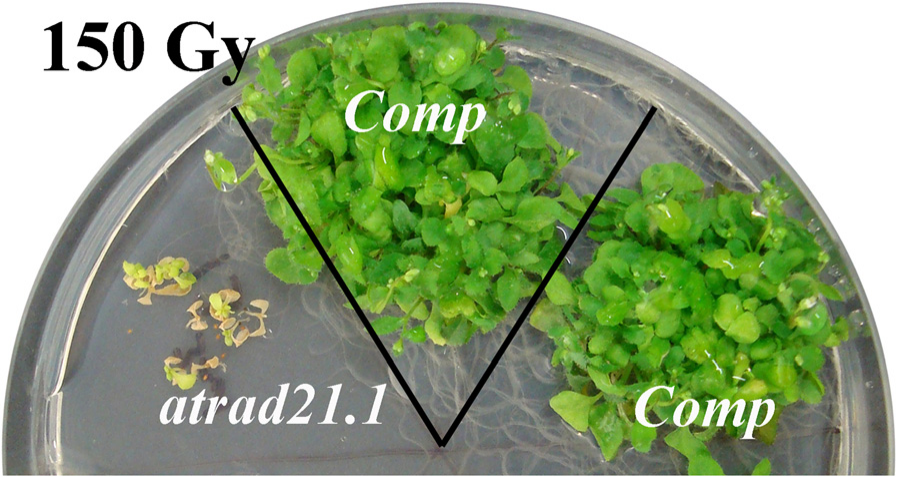
**The genomic construct, comprising the putative** ***AtRAD21.1*** **promoter region and gene, complements the** ***atrad21.1*** **mutation.** The complementation lines (*Comp*) are not hypersensitive to DNA dsb damage inducing ionising radiation, unlike the *atrad21.1* mutant. Plants were photographed 27 days after exposure of the imbibed seeds to ionising radiation (150 Gy; 3.25 Gy/minute; source: Cs137). Two independent complementation lines (*Comp*) (in *atrad21.1* mutant background carrying the complementation construct) and the *atrad21.1* mutant (with no complementation construct) are shown.

The transformation of *atrad21.1* homozygous plants with the complementation construct yielded, at least, nine independently transformed complementation lines (Comp). Five of these lines were further analysed and shown to rescue the *atrad21.1* mutant phenotype, exhibiting wild-type-like resistance to a dose of 150 Gy (3.25 Gy/minute; source: Cs137) of ionising radiation (Figure 1). These plants were genotyped and confirmed to carry the complementation construct and the *atrad21.1* mutant allele (data not shown). Hence, our results show that the *AtRAD21.1* gene and its upstream sequence are required and sufficient to rescue the *atrad21.1* mutant phenotype (hypersensitivity to ionising radiation) (Figure 1). Molecular characterisation of a Comp line exposed to ionising radiation also suggests a correlation between the re-established Col-like resistance to ionising radiation and the high amounts of *AtRAD21.1*-*GFP*-6x*His* transcript detected (Additional file 1: Figures S1a and S1c); (primer pairs: CR1 + GFPOUT and 3HOM6 + GFPOUT; Additional file 1: Table S1).

The complementation lines also demonstrate that the *atrad21.1* mutant retains the ability to be transformed and integrate T-DNA into its genome and that the epitope-tag (GFP-6xHis) fused to the predicted C-terminal end of the *AtRAD21.1* protein does not affect the function of the AtRAD21.1 protein in γ-ray irradiated imbibed seeds (Figure 1). Unfortunately, we were not able to detect GFP signal using fluorescence microscopy, either in non-irradiated or in γ-ray irradiated complementation lines (data not shown), possibly due to conformational changes of the GFP tag in the context of the recombinant protein.

### *AtRAD21.1* expression: an emergency-like response to DNA dsb damage induction

It has been shown that the transcription of *AtRAD21.1* is responsive to the induction of DNA dsb damage (in an ATM dependent manner) [14,20,21], and that the *atrad21.1* mutant imbibed seeds are hypersensitive to DNA dsb damage [14]. This suggests that the *AtRAD21.1* transcript content increase, induced by DNA dsb, may be required for DNA dsb damage repair.

It has been reported that, 1 hour after the exposure to 100 Gy of ionising radiation, no significant change in *AtRAD21.2* and *AtRAD21.3* gene transcription is detectable in a northern blot [14]. Yet, it is not known whether transcription also remains unchanged when higher doses of ionising radiation are applied and more DNA damage is induced. The *AtRAD21.2* and *AtRAD21.3* gene transcription dynamics at different time points after the induction of DNA dsb damage are also unknown. Hence, due to the importance of the RAD21 cohesin in DNA repair, and due to the lack of a more detailed characterisation of *Arabidopsis AtRAD21* gene expression responsiveness to DNA dsb, we monitored the dynamics of *AtRAD21.1*, *AtRAD21.2* and *AtRAD21.3* transcript content at different time points, during the first 48 hours After Exposure to Ionising radiation (AEI). The *AtRAD21* genes’ transcript content variation was monitored in rosette leaves from four weeks old Col plant, using quantitative real-time PCR (qRT-PCR), after exposure to 316 Gy of ionising radiation (2.65 Gy/minute; source: Co60).

**Figure 2:**
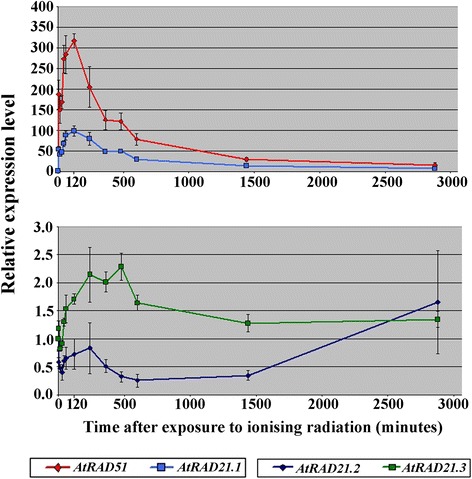
***AtRAD21.1*** **has an emergency-like transcription response to DNA dsb damage.** Steady-state *AtRAD21.1* and *AtRAD51* transcript levels increase abruptly immediately after the end of irradiation exposure (AEI) in four weeks old Col rosette leaves irradiated with 316 Gy (2.65 Gy/minute; source: Co60); Non-irradiated samples were used as reference (i.e. 1 fold). The *AtRAD21.1* and *AtRAD51* steady-state transcript level peak is detected 1 to 2 hours (AEI) (60 to 120 minutes); peaks of *circa* 100-fold and 317-fold increase in *AtRAD21.1* and *AtRAD51*, respectively. *AtRAD21.1* steady-state transcript levels revert to normal expression levels after 48 hours (2880 minutes) AEI. *AtRAD21.2* and *AtRAD21.3* transcript levels variation is mild, in comparison to AtRAD21.1, even if *AtRAD21.3* transcript steady-state levels increase by two-fold in response to DNA dsb. Values are the mean of three biological replicates for each time point. The relative transcript content was calculated using *Actin2* and *AtEF1αA4* as the reference genes, and normalized against the non-irradiated sample. The error bars represent the standard deviation. Quantitative RT-PCR data is available in Additional file 1: Table S2.

As early as 5 minutes AEI, we observed a 50-fold increase of *AtRAD21.1* transcript content in irradiated *versus* control (non-irradiated) samples (Figure 2; Additional file 1: Figures S2(A) and S2(B); Additional file 1: Table S2). The amount of transcript peaked *circa* 1 to 2 hours AEI, being almost 100-fold higher than in non-irradiated samples (Figure 2; Additional file 1: Table S2). At 4 hours AEI, the steady state levels of *AtRAD21.1* transcript progressively decrease, approaching non-irradiated levels after 48 hour AEI (Figure 2). The presented data was obtained from three independent replicates, and using two different primer pairs (Additional file 1: Table S3; primer pairs ‘1’ and ‘1 m’) targeting two different regions of the *AtRAD21.1* transcript (Additional file 1: Figure S1f).

*AtRAD51*, a gene involved in HR [17], and *AtRAD21.1* have very similar patterns of transcript steady-state content variation. This variation is, however, much more pronounced in *AtRAD51* than in *AtRAD21.1. AtRAD51* reaches a peak of 317-fold increase in transcript steady-state levels, 2 hours AEI (Figure 2; Additional file 1: Figures S2(A) and S2(B); Additional file 1: Table S2).

Reports on *AtRAD21.2* and *AtRAD21.3* gene expression after DNA dsb induction are limited to certain time points (i.e. 1 hour AEI and 1.5 hours AEI; northern blots and microarray data, respectively [14,21]), and suggest that the expression of these genes is not responsive to the induction of DNA dsb. Our results show that *AtRAD21.2* transcript content is diminished during most of the period of 48 hours after the induction of DNA dsb (Figure 2); The *AtATM* mRNA content variation after the induction of DNA dsb is more difficult to interpret since a decrease as well as an increase in transcription content is detected (Additional file 1: Figure S2(A)). In contrast, the qRT-PCR data shows that the steady-state *AtRAD21.3* transcript levels double after the exposure to 316 Gy of ionising radiation. *AtRAD21.3* expression, which is not as responsive as *AtRAD21.1* is to DNA dsb induction, reaches its peak between 4 and 8 hours AEI in contrast with *AtRAD21.1* transcript levels that reach their peak *circa* 1 to 2 hours AEI (Figure 2; Additional file 1: Figure S2(A)). These observations suggest that these two cohesin genes may be required for different roles in the cell since the dynamics of their RNA content variation, after the induction of DNA dsb damage, is not identical.

### AtRAD21.3, in association with AtRAD21.1, confers resistance to ionising radiation-induced damage

Because qRT-PCR data shows that the induction of DNA dsb induces the doubling of the *AtRAD21.3* steady-state transcript content, we investigated further if AtRAD21.3 does play a role in DNA dsb repair. Unlike *atrad21.1*, the *atrad21.3* single mutant does not exhibit clearly discernible DNA dsb damage hypersensitivity phenotypes (such as DNA damage induced lethality) [14]. Hence, we used the *atrad21.1 atrad21.3* double mutant to more easily identify and characterise the role played by AtRAD21.3 in DNA dsb. The rational is that any *atrad21.3* induced DNA dsb damage phenotype (that may go unnoticed in the *atrad21.3* single mutant because it is masked by the function played by AtRAD21.1) will be more easily detected in the double mutant. The *atrad21.1 atrad21.3* double mutant plants are viable and fully fertile, producing a full seed set in each silique (data not shown).

**Figure 3:**
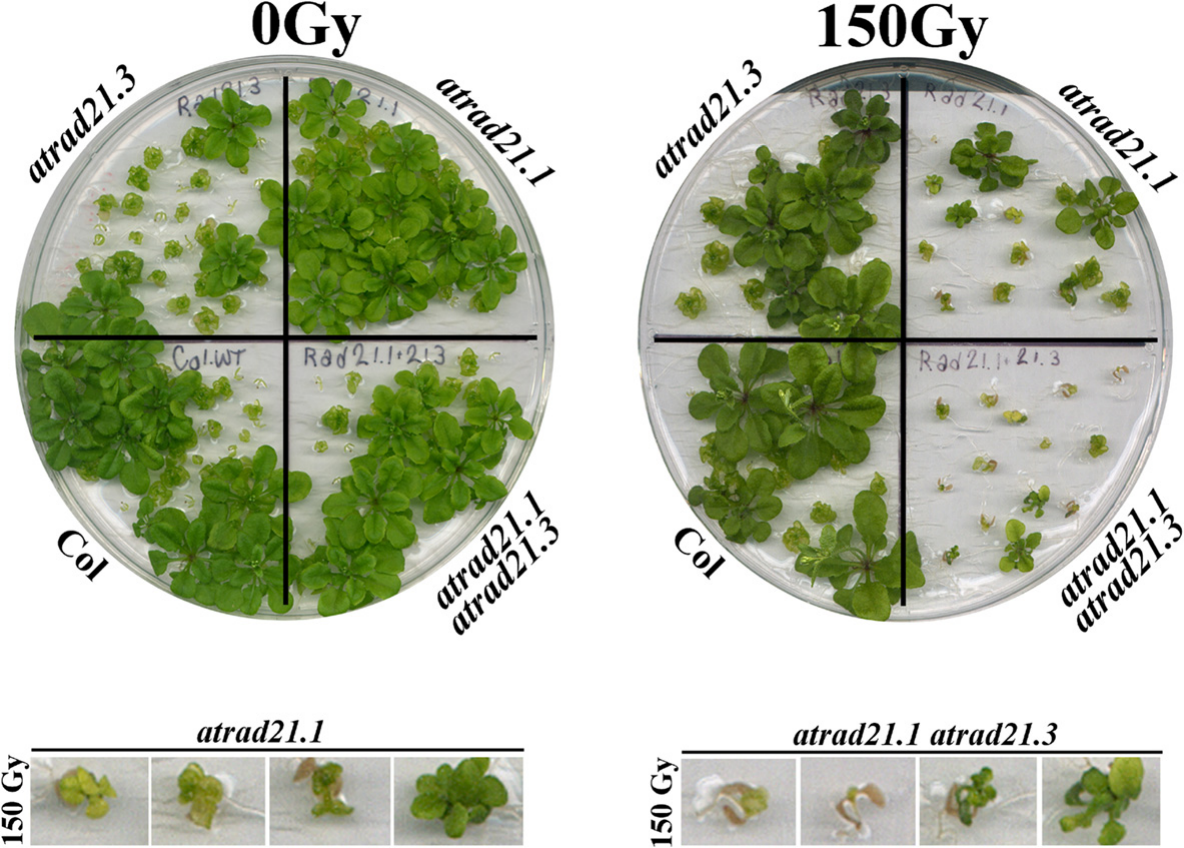
**The** ***atrad21.1 atrad21.3*** **double mutant is more hypersensitive to DNA dsb damage than** ***atrad21.1*** **.** Both the *atrad21.1 atrad21.3* double mutant and the *atrad21.1* single mutant are hypersensitive to exposure to ionising radiation (150 Gy), being the former more hypersensitive than the latter; as observed in different experimental replicas. In contrast, the *atrad21.3* single mutant reaches a development stage more similar to Col, even after exposure to 150 Gy of ionising radiation. The differences in development are highlighted in the blown up images (3× magnification) of seedlings after exposure to 150 Gy of ionising radiation. These illustrate the predominant double mutant seedlings’ phenotype; development arrest and senesce at an early developmental stage, namely in seedlings with none or one true leaf. These blown up images also show that *atrad21.1* seedlings experience severe development delay, yet not as severe as in the double mutant (seedlings bear more true leaves than the double mutant). In both the single and double mutants, some plants manage to develop further, forming more true leaves. All seedlings were germinated from irradiated imbibed seeds exposed to 150 Gy of γ-rays (0.7532 Gy/minute +/− 0.003 Gy/minute; source: Cs137) and photographed 30 days after. 0 Gy - not exposed to ionising radiation. Col - wild-type Columbia-0 plants.

**Figure 4:**
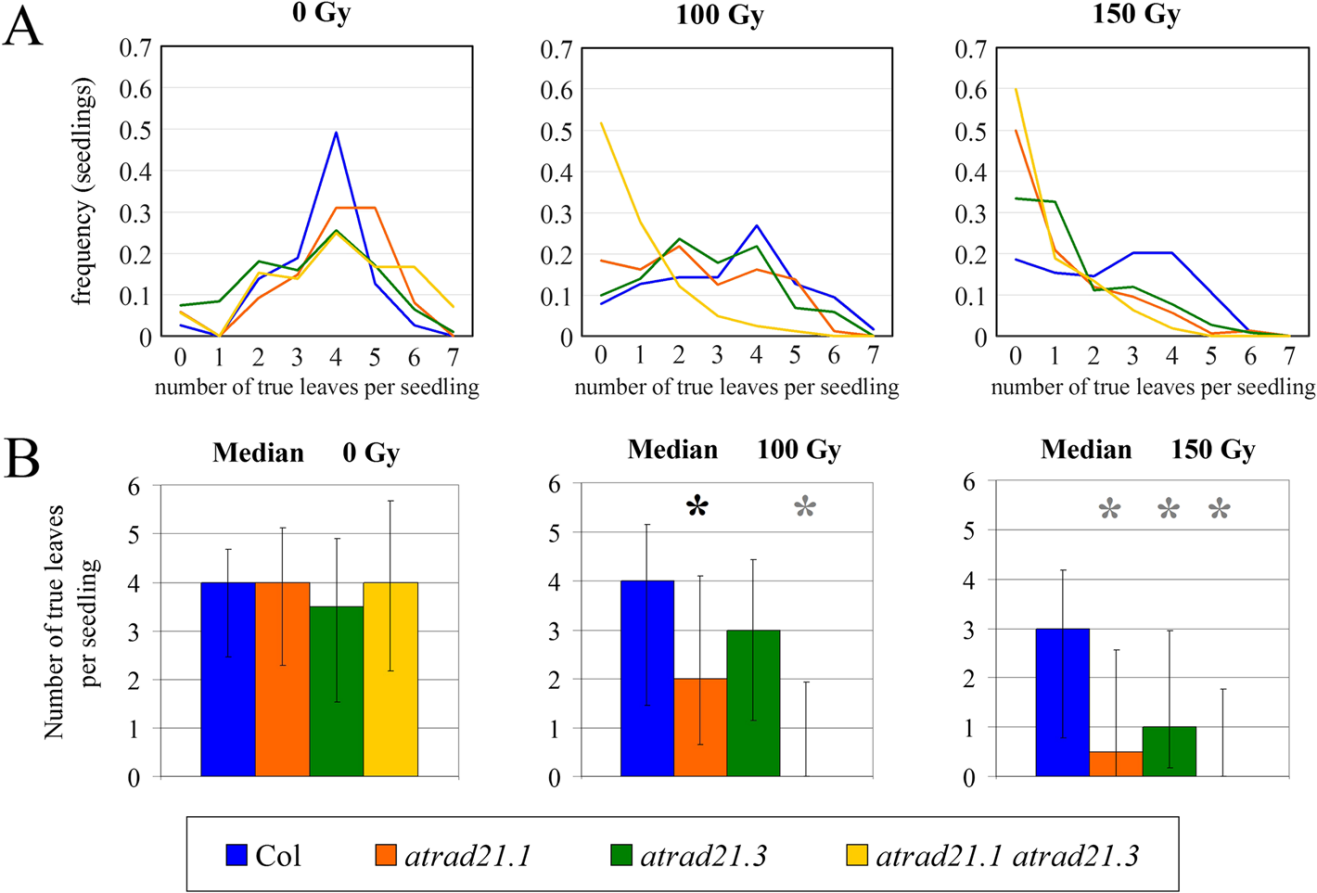
**DNA dsb severely affects development in the** ***atrad21.1 atrad21.3*** **double mutant. (A)** *atrad21.1 atrad21.3* displays the severest DNA dsb damage induced developmental arrest. The highest frequency of seedlings arrested at the early stages of development (0 and 1 true leaf) in the *atrad21.1 atrad21.3* double mutant illustrates its high hypersensitivity to DNA dsb damage. At 100 Gy, this frequency is higher in the double mutant than in the single mutants and Col; only at 150 Gy does this frequency, in *atrad21.1* and the double mutant, become similar. At 100 Gy, the frequency of seedlings with 0 and 1 true leaf, in Col and in *atrad21.3*, is similar; but at 150 Gy it becomes higher in *atrad21.3*. **(B)** *atrad21.1 atrad21.3* and *atrad21.1* are significantly different from Col (100 Gy). Medians and the Mann-Whitney non-parametric test (p value (p)<0.01, 2-tailed hypothesis) show that DNA dsb induces severe development arrest in *atrad21.1 atrad21.3*, and less so in *atrad21.1*. Both mutants are significantly different from Col (100 Gy and 150 Gy). Only at 150 Gy is *atrad21.3* also significantly different from Col. Error bars: standard deviation of the data (to the median). Black asterisk: significant difference (0<p<0.01), (Col *versus atrad21.1*; 100 Gy; U=2026; p=0.00652). Grey asterisk: significant difference (p=0) at 100 Gy: (Col *versus atrad21.1*; U=726.5); and at 150 Gy: (Col *versus atrad21.1*; U=5278.5), (Col *versus atrad21.3*; U=4712), (Col *versus atrad21.1 atrad21.3*; U=2920.5). *atrad21.3* is not significantly different from Col at 100 Gy (U=2635; p=0.06432). Figure 4 **(A** and **B)**: true leaves were counted in GM germinated seedlings, 15 days after the irradiation of imbibed seeds with 0 Gy (mock irradiation) or 100 Gy or 150 Gy (γ-rays; 3.25 Gy/minute; source: Cs137). Col - wild-type Columbia-0. Frequencies and medians were calculated with the data from the compiled data tables (Additional file 1: Table S4).

30 days after irradiating (with γ-ray) imbibed seeds with 150 Gy (3.25 Gy/minute; source: Cs137), *atrad21.1 atrad21.3* seedlings exhibit a more acute hypersensitivity to γ-irradiation than the *atrad21.1* seedlings (Figure 3). The *atrad21.1 atrad21.3* γ-ray hypersensitivity phenotype, in comparison to the *atrad21.1* and *atrad21.3* single mutants’, is characterised by a higher incidence of seedlings that bear only two expanded cotyledons and no true leaves (Figure 4). This is particularly evident at 100 Gy (γ-rays; 3.25 Gy/minute; source: Cs137) (Figure 4(A); Additional file 1: Table S4 and Figure S3), although a few seedlings do develop more true leaves. The higher incidence of seedlings with no true leaves in the *atrad21.1 atrad21.3* double mutant, in comparison to the *atrad21* single mutants and Col, is clearly reflected in the value of the medians (Figure 4(B)), modes and means (Additional file 1: Table S5 and Figure S4). Furthermore, according to the Mann–Whitney U-test analysis of the number of true leaves data (obtained 15 days after the exposure to 100 Gy and 150 Gy (γ-rays; 3.25 Gy/minute; source: Cs137)), the *atrad21.1 atrad21.3* double mutant is significantly different (p value (p) =0, 2-tailed hypothesis) from Col (Figure 4(B)). Comparatively to the double mutant γ-ray hypersensitive, *atrad21.1* mutant seedlings bear more true leaves. Still, *atrad21.1* is developmentally delayed in comparison to wild-type as far as the number of true leaves and the size of the leaves is concerned (Figures 3 and 4). At 100 Gy, *atrad21.1* is already significantly different from Col, albeit with a higher p value (p = 0.00652) than the double mutant (p = 0). In contrast, *atrad21.3* is not significantly different from Col at 100 Gy (p = 0.06432). Only at 150 Gy is it possible to detect a significant difference between *atrad21.3* and Col (Figure 4(B); Additional file 1: Figure S4). Ultimately, many, if not all, of the seedlings exhibiting hypersensitivity to ionising radiation (mostly the *atrad21.1* and the *atrad21.1 atrad21.3* mutants with none or few true leaves) will senesce.

### The kinetics of DNA dsb damage repair is affected, and higher basal levels of DNA dsb are detected, in the *atrad21.3* mutant

To further characterise the role of AtRAD21 cohesins, we monitored repair of DNA dsb by comet assays in 10-days-old seedlings exposed to Bleomycin. We chose to use Bleomycin, a radiomimetic cancerostatic agent that induces DNA dsb in a similar manner to ionising radiation [39], because it allowed us to compare our results with previously published data of DNA dsb repair kinetics [23,40,41]. Three different *atrad21* homozygous mutants were used in the comet assay (*atrad21.1*, *atrad21.3* and *atrad21.1 atrad21.3*). The *atrad21.2* mutant was excluded from this and other assays because, to the best of our knowledge, there are no viable *atrad21.2* homozygous mutant knockout lines available [42].

**Figure 5:**
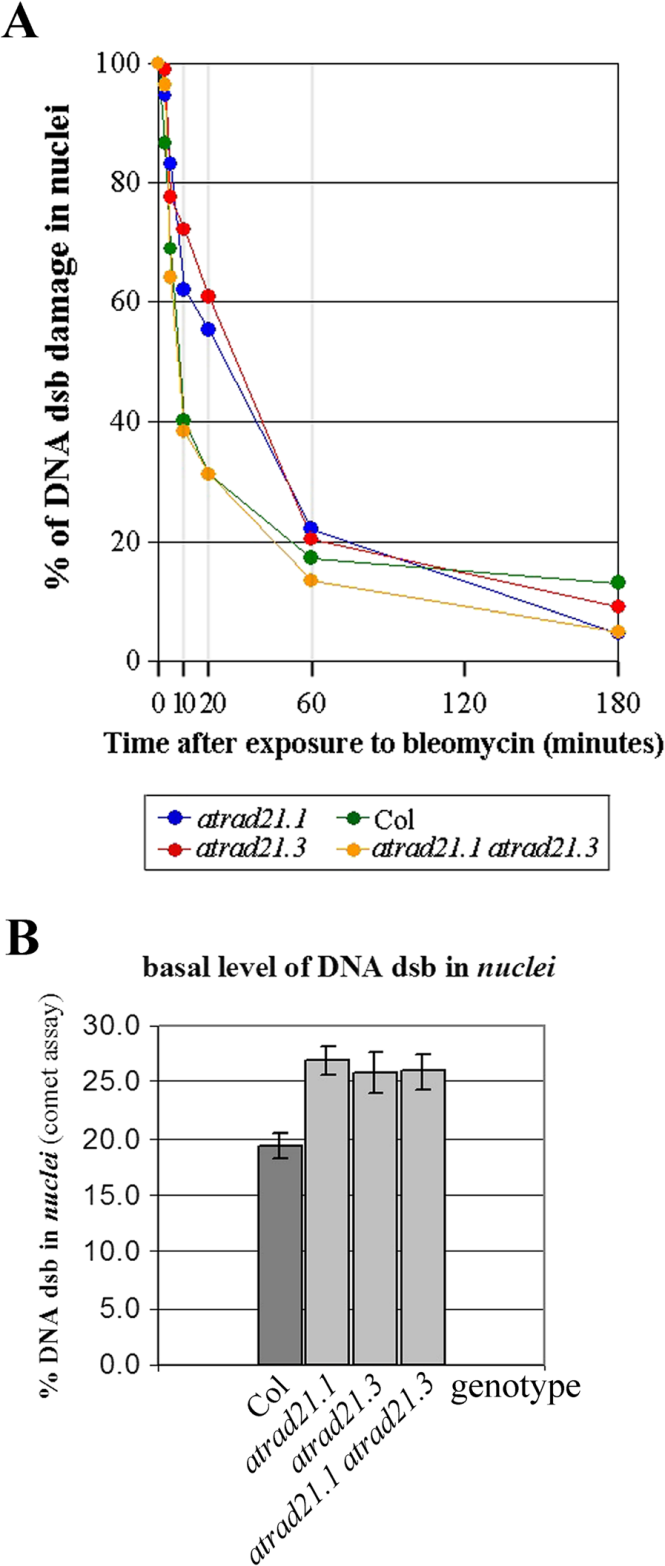
**DNA dsb basal levels and repair kinetics are altered in the atrad21.1 and atrad21.3 mutants. (A)** *atrad21.1* and *atrad21.3* single mutants’ DNA dsb damage repair kinetics is similar. During the first 60 minutes after DNA dsb damage induction, *atrad21.1* and *atrad21.3* mutants retain more unrepaired DNA dsb than Col. This difference is more striking at 10 minutes (62.1% to 72.2% of induced DNA dsb remain unrepaired in the single mutants *versus* 40.2% in Col), 20 minutes (55.4% to 60.9% in the single mutant *versus* 31.3% in Col) and 60 minutes (20.3% to 22.1% in the single mutants *versus* 17.1% in Col) after DNA dsb damage induction. The *atrad21.1 atrad21.3* double mutant has a Col-like DNA dsb damage repair kinetics. DNA dsb damage quantification was carried out on *nuclei* from 10-days old seedlings harvested 0, 3, 5, 10, 20, 60 and 180 minutes after exposure to 30 μg/ml Bleomycin. Col - wild-type Columbia-0 plant. **(B)** *atrad21.1*, *atrad21.3* and *atrad21.1 atrad21.3* mutants have higher basal levels of DNA dsb than Col. The amount of DNA dsb detected, by comet assay, in *nuclei* obtained from seedlings not exposed to DNA dsb inducing agent, indicates that the amount of DNA dsb detected in Col is significantly lower than the amount detected in *atrad21.1*, *atrad21.3* and *atrad21.1 atrad21.3* mutants. DNA dsb damage quantification was carried out on *nuclei* from 10-days old seedlings that were not exposed to Bleomycin. Error bars represent the standard error. Col - wild-type Columbia-0 plant. Comet assay data is available in Additional file 1: Table S6.

Repair kinetics observed in seedlings of wild-type Col, *atrad21.1*, *atrad21.3* and the *atrad21.1 atrad21.3* double mutant control (not exposed) and exposed to 10 μg/ml Bleomycin are not significantly different (data not shown). However, when higher Bleomycin concentrations (30 μg/ml) are used, which result in the induction of more DNA dsb [40], impaired DNA dsb repair becomes perceptible in the single mutants relative to wild-type. Significant differences are particularly evident between 10 to 60 minutes after DNA dsb induction (Figure 5(A)), i.e. in the transition period from the initial fast phase of dsb repair kinetics to the following slow phase of dsb repair kinetics [43,44] (Additional file 1: Figure S5; Additional file 1: Table S6). Unlike the single mutants, *atrad21.1 atrad21.3* has wild-type-like (Col-like) DNA dsb damage repair kinetics when exposed to 30 μg/ml Bleomycin. Yet, the double mutant as well as the *atrad21.1* and the *atrad21.3* single mutants exhibit a significantly higher content of nuclear DNA dsb (high basal level of DNA dsb) then the wild-type (Figure 5(B)), even when there is no induction of DNA dsb.

### The *atrad21.1 atrad21.3* double mutant hypersensitivity to DNA dsb damage is less acute than in the *atku80 atrad21.1* double mutant and the *atku80* single mutant

DNA dsb are repaired via different DNA repair pathways. RAD21 has been proposed to facilitate DNA dsb repair via HR by keeping the homologous DNA sequences of sister chromatids in close proximity [7]. However, in plants, DNA dsb are predominantly repaired via direct joining of double stand breaks’ ends (particularly via the Canonical-Non-Homologous-End Joining – C-NHEJ), which do not require an extended homologous DNA sequence strand for repair [45]. To determine the consequences of disrupting AtRAD21 (which has been associated with HR) in a C-NHEJ DNA repair pathway mutant, we’ve introgressed the *atrad21.1* mutant allele into the *atku80* mutant background [46] to produce the *atrad21.1 atku80* double mutant.

**Figure 6:**
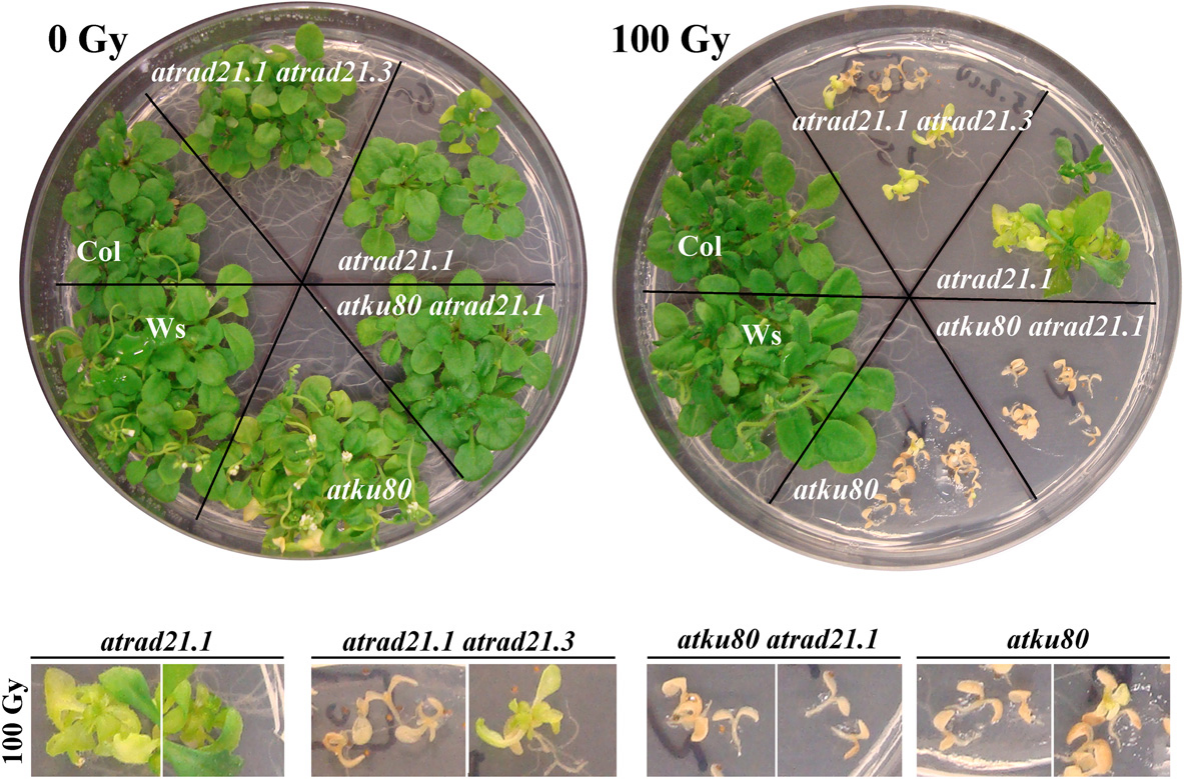
**C-NHEJ** ***versus*** **HR associated** ***atrad21*** **DNA dsb damage hypersensitivity.** Comparison of the DNA dsb damage induced phenotypes of the C-NHEJ associated *atku80* mutant *versus* the HR associated *rad21* mutations. Imbibed seeds of mutants homozygous for the *atku80* mutant allele (*atku80 atrad21.1* and *atku80*) are extremely hypersensitive to DNA dsb; furthermore, they are more hypersensitive to DNA dsb than the *atrad21.1 atrad21.3* double mutant and even more so than the *atrad21.1* single mutant; this has been confirmed in different experimental replicas. The blown up (2× magnification) seedlings’ pictures show *atku80* and *atku80 atrad21.1* exhibiting a more severe hypersensitivity to DNA dsb damage than *atrad21.1 atrad21.3*. While some *atrad21.1 atrad21.3* seedlings are still able to form some true leaves (a seedling with nine small true leaves is shown) after irradiation with 100 Gy of ionising radiation, *atku80* and *atku80 atrad21.1* development is arrested at an earlier stage (seedlings with no true leaves or with one incipient true leaf). The *atrad21.1* seedlings exhibit the least hypersensitive phenotype of all four mutants. Imbibed seeds were exposed to 100 Gy of ionising radiation (3.25 Gy/minute; source: Cs137); 0 Gy were not exposed to ionising radiation. The seedlings germinated from the irradiated imbibed seeds were photographed 23 days after the exposure to ionising radiation. Col - wild-type Columbia-0 plant; Ws - wild type Wassilewskija-1 plant.

The *atku80 atrad21.1* double mutant plants were genotyped (Additional file 1: Figure S6) and shown to be viable. Under normal growth conditions (non-irradiated with ionising radiation), these plants have a normal vegetative and fertility phenotype; seed set in each silique of the double mutant is indistinguishable from that of Col plants (data not shown). When the imbibed seeds of *atku80 atrad21.1* double mutant, and the *atku80* mutant, are exposed to γ-rays (100 Gy, 3.25 Gy/minute; source: Cs137), both mutants exhibit a similar acute hypersensitivity phenotype (Figure 6; 100 Gy). No hypersensitivity to DNA dsb is detected when imbibed Col, *atku80* and *atku80 atrad21.1* mutant seeds are irradiated with 50 Gy (3.25 Gy/minute; source: Cs137) of ionising radiation (data not shown).

Comparison of hypersensitivities to DNA dsb induced by ionising radiation shows that *atku80* and *atku80 atrad21.1* mutants are clearly more hypersensitive to DNA dsb than the *atrad21.1 atrad21.3* double mutant, and even more so then the *atrad21.1* single mutant (Figure 6).

These observations indicate that even though the AtRAD21.1 and AtRAD21.3 cohesins play an important role in DNA dsb repair in imbibed seeds, the AtKu80 protein, that is associated with C-NHEJ, plays a predominant role in DNA dsb repair. This is in agreement with previous reports that show that the C-NHEJ repair pathway is the predominant repair pathway in plants [45]. Due to the severity of the *atku80* and *atku80 atrad21.1* mutant phenotypes it is not possible to determine if the DNA damage hypersensitivity phenotype observed in the *atku80 atrad21.1* double mutant is identical to that of the *atku80*, or if it is cumulative, yet masked by the severity of the *atku80* phenotype.

## Discussion

### *AtRAD21.1* and *AtRAD21.3 Arabidopsis thaliana* cohesins’ emergency response to DNA dsb damage

The increase of steady-state *AtRAD21.3* RNA levels, and more dramatically, the rapid and immediate increase of steady-state *AtRAD21.1* RNA levels after the induction of DNA dsb suggests that both cohesins play a role in an emergency response to DNA dsb damage (Figure 2; Additional file 1: Figure S2). Transcription upregulation of the *AtRAD21.1*-*GFP*-6x*His* transgene in the complementation line plants upon exposure to ionising radiation (Additional file 1: Figure S1c) and the rescue of the *atrad21.1* DNA dsb damage hypersensitivity phenotype in these same lines (Figure 1) links the *AtRAD21.1* emergency response to DNA dsb repair. This data suggests that the upregulation of *AtRAD21.1* transcriptional activity could be directly correlated with an increase in cohesion induced by DNA dsb (*de novo* cohesion). This hypothesis is in accordance with the reported observation that DNA damage induces in Col an increase in sister chromatid cohesion just 10 minutes after exposure to irradiation [47]. Moreover, and also 10 minutes after the induction of DNA dsb damage, the *atrad21.1* mutant experiences a striking delay in DNA dsb repair (Figure 5(A)). Together, these observations suggest that, as observed with RAD21 homologues in other organisms, AtRAD21.1 could also be involved in DNA dsb induced *de novo* cohesion required for DNA dsb repair in Arabidopsis. Indeed, in yeast and human cells, it has been proposed that the recruitment of RAD21 cohesin to chromosomes after DNA dsb induction [7,9,10] reinforces the tethering of sister chromatids by quickly establishing DNA dsb induced *de novo* cohesion. Further experiments will be required to demonstrate if this AtRAD21.1 emergency response indeed leads to the *de novo* cohesion and the increased sister chromatid cohesion. The upregulation of *AtRAD21.3* transcription (Figure 2) and the concurrent slower DNA dsb repair detected 10 minutes after the induction of DNA dsb (Figure 5) suggest that AtRAD21.3 may also be involved in an AtRAD21.1-like DNA dsb repair emergency response.

Finally, the similar timing of *AtRAD51* and *AtRAD21.1* transcript content increase (qRT-PCR data) suggests that AtRAD21.1 might also be required during the first stages of DNA dsb repair. AtRAD51, similarly to its homologues in yeast, is thought to be involved in DNA strand invasion and homology search during the first stages of recombination [48-50]. Hence, AtRAD21.1 may play a role at the early stages of somatic recombination (DNA dsb repair) too.

### Both AtRAD21.1 and AtRAD21.3 are required for DNA dsb repair

AtRAD21.1 and AtRAD21.3 are required for DNA dsb repair when numerous DNA dsb are induced (30 μg/ml Bleomycin) (Figure 5(A)), as well as when plants are not exposed to DNA dsb inducing agents (Figure 5(B)).

*atrad21.1*, *atrad21.3 single* mutants, and the *atrad21.1 atrad21.3* double mutant, exhibit similar and significantly higher basal level of DNA dsb when compared to Col (Figure 5(B)). This indicates that AtRAD21.1 and AtRAD21.3 are probably required for the repair (or restrict the formation) of DNA dsb induced by endogenous stresses (such as DNA replication) or naturally occurring environmental stresses.

DNA repair kinetics data from *atrad21.1* [40], *atrad21.3* (Figure 5(A)), and other Arabidopsis mutants affecting HR in somatic tissue and with no apparent deleterious defects during meiosis, such as *atrad17* [26] and *gmi1* (a SMC-Hinge Domain containing protein) [23], shows that these mutants experience a delay in DNA dsb repair when many DNA dsb are induced. This delay is evident, as early as 10 to 20 minutes after bleomycin treatment, in the *atrad21.1* and *atrad21.3* mutant seedlings (Figure 5(A)), as well as in the *gmi1* mutants [23]. These similarities suggest that like GMI1, AtRAD21.1 and AtRAD21.3 may also be involved in HR. The decrease in DNA dsb repair kinetics observed in the *atrad21.1* and *atrad21.3* single mutants has also been observed in yeast strains that contain low amounts of RAD21 protein [51]. This suggests a correlation between the amount of induced DNA dsb in the cell, and the amount of RAD21 protein required for the DNA dsb repair. Indeed, when low Bleomycin concentration (10 μg/ml) is used, inducing few DNA dsb, the observed repair kinetics between Col, the *atrad21.1*, *atrad21.3* and *atrad21.1 atrad21.3* is not significantly different (data not shown). One possible explanation for the similarity in repair kinetics being that the level of chromosome cohesion remaining in the mutant lines is sufficient to countervail the small amount of DNA dsb produced, and hence the efficiency of DNA dsb repair is not affected. Yet, when more DNA dsb are produced (30 μg/ml Bleomycin) [40], the lack of AtRAD21.1 or AtRAD21.3 in the single mutants becomes critical for DNA repair.

We hypothesise that an increasing number of DNA dsb in the cell leads to an increasing need of an abundant pool of cohesin proteins to establish *de novo* cohesion, to allow DNA dsb HR repair. Hence, a less abundant pool of cohesins in the *atrad21.1* and *atrad21.3* single mutants would account for the less efficient DNA repair (slower kinetics) observed during the first 10 to 20 minutes after the induction of a high incidence of DNA dsb (Figure 5(A)). The slower DNA repair kinetics observed in the *atrad21.1* and *atrad21.3* single mutants could also be caused by an AtRAD21-dependent DNA-damage-repair-checkpoint. Indeed, the yeast *rad21* mutation has been correlated with the disruption of DNA-damage-induced-checkpoints. Likewise, in mammalian cells, RAD21 is involved in DNA-damage-induced cell cycle progression arrest during DNA replication and at the G2/M cell cycle stages [52-54].

In the particular case of the *atrad21.1 atrad21.3* double mutant, which has a wild-type-like repair kinetic (Figure 5(A)), it is plausible that due to the knockout of both *AtRAD21.1* and *AtRAD21.3* genes (Additional file 1: Figure S6), an AtRAD21-dependent DNA dsb repair pathway becomes fully compromised. Consequently, we propose that in the double mutant, the DNA dsb repair is switched to an AtRAD21-non-dependent DNA dsb repair pathway with a kinetics similar to the one observed in the *atku80* mutant [40] and wild-type. AtKu80 is associated with NHEJ DNA repair, unlike RAD21 (the AtRAD21 homologue), AtRAD17 and GMI that are associated with HR [23,26,44,53,55].

Further experiments will help validate these or other hypothesis.

### Acute hypersensitivity to DNA dsb in the *atrad21.1 atrad21.3* double mutant

Despite the lack of AtRAD21.3 protein, which has been attributed a role in sister chromatid arm cohesion and centromere cohesion [37], the *atrad21.3* single mutant morphology appears not to differ from that of Col, after exposure to ionising radiation (Figure 3). Only a more detailed characterisation (number of true leaves) of the *atrad21.3* mutant indicates that only after exposure to high doses of radiation (Figure 4(B); 150 Gy) does the difference between *atrad21.3* and Col becomes significant. Furthermore, the lack of AtRAD21.3 cohesin in the *atrad21.1 atrad21.3* mutant background results in a higher DNA dsb hypersensitivity phenotype, comparatively to the *atrad21.1* single mutant’s and Col’s DNA dsb hypersensitivity phenotype (Figure 3; Figure 4; Figure 6). These results indicate that both AtRAD21.1 and AtRAD21.3 contribute to the plant’s ability to cope with DNA dsb damage, with AtRAD21.3 having a synergistic and non-redundant effect on the AtRAD21.1 function. Other examples exist of synergistic actions on DNA dsb damage repair and genome stability, namely *AtRAD50* and *TERT*, *NBS1* and *TERT*, *NBS1* and *ATM* [25,56,57].

A shift from an AtRAD21-dependent, possibly error free HR repair, to an error-prone-AtRAD21-independent DNA dsb repair pathway could be at the origin of the increased hypersensitivity of the *atrad21.1 atrad21.3* double mutant to DNA dsb damage. This shift would give rise to an increased frequency of deleterious mutations resulting from the DNA dsb repair, hence inducing the enhanced hypersensitivity to DNA dsb observed in the double mutant (Figure 2). Increased frequency of genomic lesions has been observed in the moss *Physcomitrella patens ppmre11* and *pprad50* mutants [41]. These authors propose that this increased frequency of lesions is caused by a shift to an error-prone DNA repair pathway that directly joins the DNA dsb ends after processing them, and also by the disruption of the RAD50 and MRE11 role in tethering the two DNA dsb ends in close proximity.

Even though *atrad21.1 atrad21.3*, *atku80* and wild-type have comparable DNA dsb repair kinetics, the double mutant is not as hypersensitive to DNA dsb as the *atku80* mutant [40] (Figure 6). One can speculate that this difference is caused by the choice of different DNA dsb repair pathways in the imbibed seeds of the *atku80* and *atrad21.1 atrad21.3* mutants.

### Proposed role of AtRAD21.3 and AtRAD21.1 in sister chromatid cohesion and DNA repair

**Figure 7:**
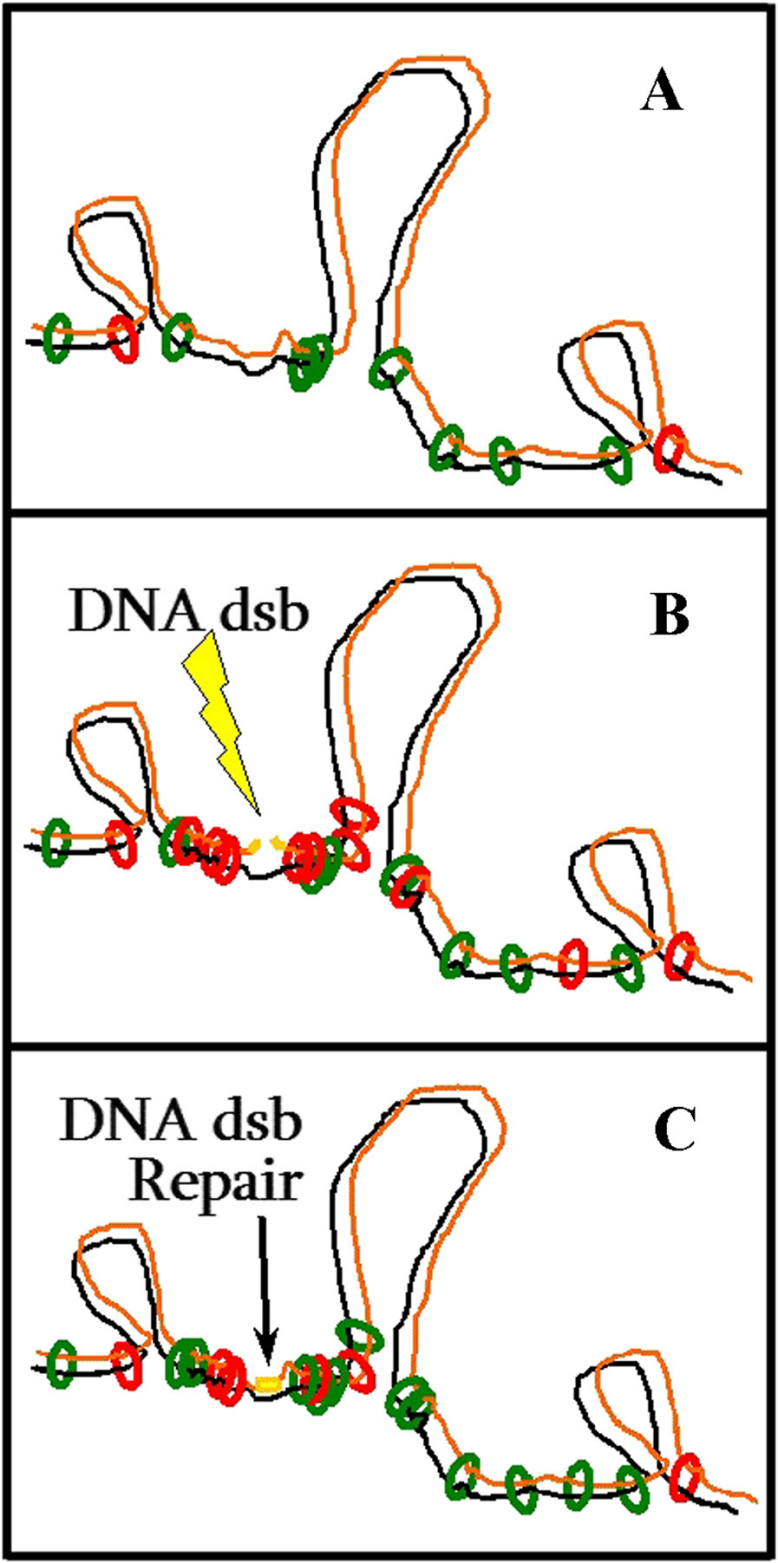
**Proposed model: AtRAD21.1 and AtRAD21.3 before and after induction of DNA dsb damage. (A)** Before the induction of DNA dsb, sister chromatid cohesion is promoted by AtRAD21.3 (green rings), and possibly also by some AtRAD21.1 (red rings) associated with DNA dsb created by endogenous factors. **(B)** After the induction of DNA dsb breaks (flash), *AtRAD21.1* expression is enhanced. This is expected to increase the pool of cohesin complexes containing AtRAD21.1 (red rings) in the cell, hence contributing to promote and enhance sister chromatid cohesion. **(C)** The DNA dsb damage induced increase of *AtRAD21.3* transcript content (that occurs after that of AtRAD21.1), is also expected to contribute to increase the pool cohesin complexes containing AtRAD21.3 (green rings). These cohesin complexes (green circles) may reinforce sister chromatid cohesion, or they may replace (all or some of) the AtRAD21.1 cohesin complexes (red rings) that generated the *de novo* cohesion. It has been proposed that the increased cohesion facilitates DNA dsb repair by promoting physical proximity between the chromatid with a DNA dsb (orange) and its intact sister chromatid (black). Green and red rings: cohesin complexes tethering the two sister chromatids (black and orange). Yellow lines: the site of the DNA dsb. Flash (yellow): the DNA dsb inducing agent.

In this work we show that both AtRAD21.3 and AtRAD21.1 are involved in DNA dsb repair. In Figure 7 is illustrated an hypothesis that proposes that upon induction of DNA dsb, the *AtRAD21.1* emergency transcriptional response ensures an enriched pool of AtRAD21.1 that will reinforce sister chromatid cohesion after the induction of DNA dsb. This function appears to be required at the early stages of DNA dsb repair (Figure 2), and is crucial since the *atrad21.1* is hypersensitive to DNA dsb damage. *AtRAD21.3* upregulation is also proposed to contribute, but at a later stage, to the pool of AtRAD21 cohesin proteins required for DNA dsb repair after the induction of DNA dsb. However, the AtRAD21.3 primarily role may be to establish chromosome cohesion and contribute to chromosome structure regardless of the presence or the absence of DNA dsb. Indeed, data from Takahashi and Quimbaya *et al*. [36] hint that AtRAD21.3 cohesion may be associated with DNA replication. Hence, the major AtRAD21.3 contribution to the repair of DNA dsb may be to provide a pre-existing chromosome scaffold and cohesion that will aid the repair of DNA dsb that arise subsequently.

The conjecture that AtRAD21.3 plays a role in chromosome structure is based on evidences that the RAD21 protein, in metazoans, is involved in chromatin structure [58,59] and associates with the nuclear matrix [60]. Interestingly, like for the *atrad21.3* mutant [14] (Additional file 1: Figure S7), it has also been reported that the mis-expression or the knocking-out of some matrix-associated proteins that contribute to chromatin remodelling, also affects flower-bolting time [61,62].

## Conclusions

The identification of AtRAD21.3’s involvement in DNA dsb damage repair adds another player to the group of known proteins that are involved in DNA dsb repair in Arabidopsis. A role for AtRAD21.3 in DNA dsb damage repair is clearly demonstrated by the comet assay data and the γ-ray hypersensitivity phenotype observed in the *atrad21.1 atrad21.3* double mutant. Likewise, the reduced number of true leaves in the *atrad21.3* single mutant, in comparison to Col, particularly after exposure to high dosages of radiation (150 Gy), is also an indication that AtRAD21.3 plays a role in DNA dsb damage repair. Furthermore, the different γ-ray hypersensitivity phenotypes exhibited by the *atrad21.1*, *atrad21.3* and *atrad21.1 atrad21.3* mutants, and the fact that both genes are upregulated in response to DNA dsb damage indicates that their functions in DNA dsb damage repair are not redundant. Our data reveals an increased level of complexity to the involvement of cohesins in DNA dsb damage repair that could be specific to plants.

## Methods

### Plant material

*Arabidopsis thaliana* seeds were surface sterilised, plated in germination medium, and imbibed in the dark at 4°C, for three to four days. All seedlings, except those depicted in Figure 3, were grown on germination solid medium (GM) (MS medium + Gamborg B5 vitamins, 1% sucrose; 0.8% micro-agar - Duchefa) in sterile Petri dishes or in pots containing a sterilised commercial mix of turf, soil and fertiliser; pH 5.5 - 6.5. Both GM and soil grown plants were kept in growth chambers with a light cycle of 16 hours of light at 22°C alternating with 8 hours of darkness at 19°C. In the assay depicted in Figure 3, imbibed sterilised seeds were germinated and grown for 30 days in ½MS solid medium, in a growth chamber with continuous light (24 hours) at 21°C.

Primers used for genotyping are shown in Additional file 1: Table S7, and plant material sources (Col, Ws, *atku80*, *atrad21.1*, *atrad21.3* and *atrad21.1 atrad21.3*) are described in Additional file 1: Materials and Methods. Plant genomic DNA was extracted using the method devised by Edwards *et al*. [63].

### *AtRAD21.1* complementation construct

The complementation construct comprises the upstream genomic region (2,602 bp) and the coding genomic sequence (containing the introns and exons) of *AtRAD21.1* (4,109 bp), excluding the translation stop codon. The genomic fragment was PCR amplified using the Pfx50 DNA polymerase enzyme (Invitrogen), following the manufacturer’s protocol; the primers GP1G (CACCGCATCTTTGCTCACCTACCTCAAACG) and GR1cDR (ACAAGCTTTTTGTGGTCTGGAAACACGCAT) were used (Additional file 1: Figure S1f)). Genomic DNA isolated from the MHK7 P1 clone (provided by Arabidopsis Biological Resource Centre - ABRC) was used as template. The PCR product was cloned in a pENTR/D-topo vector (Invitrogen), recombined into the pMDC107 vector [64] using the LR clonase II enzyme mix reaction (Invitrogen), and sequenced.

### *AtRAD21.1* complementation lines

Col and *atrad21.1* homozygous mutant plants were transformed with the genomic *AtRAD21.1*-*GFP*-6x*His* complementation construct by the floral dip method [65], using the *Agrobacterium tumefaciens* GV3101 strain carrying the plasmid pMP90RK [66]. T1 transformants were selected on hygromycin containing media, and T2 seeds harvested. T2 seeds were exposed to ionising radiation (DNA damage sensitivity assay) and those plants not displaying hypersensitivity to DNA dsb (27 days after exposure to 150 Gy of ionising radiation) were genotyped (primer pairs information is available in Additional file 1: Table S7). The expression of the *AtRAD21.1*-*GFP*-6x*His* construct was assessed (verifying complementation) by RT-PCR using RNA extracted from samples, frozen in liquid nitrogen, 1 hour after exposure to irradiation and mock-irradiation (Additional file 1: Table S1). RNA was extracted from irradiated (150 Gy; 3.25 Gy/minute; source: Cs137) rosette leaves from a heterozygous *atrad21.1* plant, and seedlings from a complementation line (in *atrad21.1* homozygous mutant genetic background) and from non-irradiated (mock) Col rosette leaves. cDNA was synthesised (as described in Additional file 1: Materials and Methods).

### Ionising radiation sensitivity assays

Four weeks old Col plants grown *in vitro* (in GM solid medium) were irradiated with 316 Gy (2.65 Gy/minute; source: Co60), in the dark. The dosage of radiation absorbed by the samples was monitored with radiation dosimeters placed under and over the irradiated samples. After irradiation, the plants were placed back in the growth chamber.

Irradiation of seeds was carried out after surface sterilising, and imbibing the seeds in sterile 0.1% agarose for 3 to 4 days at 4°C, in the dark. Seeds were irradiated inside a GammaCell 2000 with the calculated dosages of 50 Gy, 100 Gy or 150 Gy of ionising radiation (γ-rays; 3.25 Gy/minute; source: Cs137), or inside a GammaCell 40 with the calculated dosages of 150 Gy (γ-rays; 0.7532 Gy/minute +/− 0.003 Gy/minute; source: Cs137). After irradiation, seeds were plated on GM solid medium and grown in sterile conditions in the growth chamber. Imbibed seeds and four weeks-old plants, used as experimental controls, were not irradiated (0 Gy).

### Statistical analysis

The number of emerging, and formed, true leaves was counted in individual seedling after 15 days after the irradiation (15 DAI), or mock-irradiation, of the seeds. The irradiated (and non-irradiated, 0 Gy) seeds were imbibed in 0.1% agarose for 3 days, in the dark at 4°C, prior to being irradiated with 100 Gy or 150 Gy (γ-rays; 3.25 Gy/minute; source Cs137). The seeds were germinated and grown in sterile petri dishes, on germination solid medium (GM), in a growth chamber (16 hours of light, 22°C; 8 hours of dark, 19°C).

Non-parametric Mann–Whitney U-test [67] (p < 0.01; 2-tailed hypothesis) was performed to determine if there are significant differences between the *atrad21.1*, *atrad21.3* and *atrad21.1 atrad21.3* mutants and the wild type plants Col; differences in hypersensitivity to ionising radiation exposure were estimated via the number of true leaves per seedlings. The compiled data (Additional file 1: Table S4) was used in the Mann–Whitney U-test.

### RNA extraction and quantitative real-time PCR expression data acquisition and analysis

Rosette leaves from non-irradiated and irradiated four weeks old Col plants grown in GM medium were harvested 5, 15, 30 and 45 minutes, and 1, 2, 4, 6, 8, 10, 24 and 48 hours after the 316 Gy irradiation session (2.65 Gy/minute; source: Co60), and immediately frozen in liquid nitrogen and stored at −80°C. RNA was extracted from three independent biological replicas (irradiated and non-irradiated) and cDNA was synthesised. The transcript steady-state levels were quantified by Real-Time PCR. The monitored genes were: the cohesin genes being characterised (*AtRAD21.1*, *AtRAD21.2*, *AtRAD21.3*), *AtATM*, the positive control *AtRAD51* [68], and the reference genes (*Actin2* and *AtEF1αA4*) [47,68]. Relative quantification of transcript accumulation was obtained using the Pfaffl method [69]. Primer information and further protocol information is provided in Additional file 1: Materials and Methods and Additional file 1: Table S3.

### Comet assay: DNA dsb induction, data acquisition and evaluation

Nuclear DNA dsb fragmentation of 10-days-old Arabidopsis seedlings (Col and *atrad21.1*, *atrad21.3* and *atrad21.1 atrad21.3* homozygous mutant lines) was assessed with the neutral protocol for single cell gel electrophoresis (comet) assay [40,70]. Untreated seedlings, and seedlings treated with 10 μg/ml and 30 μg/ml Bleomycin Sulfate (cancerostatic Bleomedac Medac, Germany) for 1 hour in liquid ½MS, were harvested and frozen in liquid nitrogen 3, 5, 10, 20, 60 and 180 minutes after the Bleomycin treatment (DNA dsb inducing agent). After processing, nuclear ‘comets’ were stained with SYBR Gold stain (Molecular Probes/Invitrogen), viewed in an epifluorescence Nikon Eclipse 800 microscope and evaluated by the Comet module (LUCIA Comet Assay) of the LUCIA cytogenetics software (LIM, Czech Republic). Three independent experiments were performed and compiled. The incidence of DNA dsb was measured as the fraction of fragmented DNA that moved from the comet head to the comet tail (% tail-DNA). The calculated percentage of DNA damage remaining for each given repair time t_x_ is defined as:

$$ \mathrm{K}\left(\mathrm{t}\right)=\%\ \mathrm{damage}\ \mathrm{remaining}\left(\mathrm{t}\mathrm{x}\right)\frac{\mathrm{mean}\ \%\mathrm{tail}\hbox{-} \mathrm{D}\mathrm{N}\mathrm{A}\left(\mathrm{t}\mathrm{x}\right)\hbox{-} \mathrm{mean}\ \%\mathrm{tail}\hbox{-} \mathrm{D}\mathrm{N}\mathrm{A}\left(\mathrm{control}\right)}{\mathrm{mean}\ \%\mathrm{tail}\hbox{-} \mathrm{D}\mathrm{N}\mathrm{A}\left(\mathrm{t}0\right)\hbox{-} \mathrm{mean}\ \%\mathrm{tail}\hbox{-} \mathrm{D}\mathrm{N}\mathrm{A}\left(\mathrm{control}\right)}\times 100 $$

A more detailed protocol is provided in Additional file 1: Materials and Methods.

### Supporting data

The data set(s) supporting the results of this article are included within the article and in its additional file.

## Additional file

Additional file 1:
**Materials and Methods Plant Material; qRT-PCR analysis; Comet assay.** **Figure S1.** *AtRAD21.1*-*GFP*-6x*His* transcript detection, and gene schematic representation. **Figure S2.** Relative variation of steady-state transcript levels during the 48 hours after the exposure to ionising radiation. **Figure S3.** Frequency of seedlings with different numbers of true leaves, in different genotypes, before and after exposure to ionising radiation. **Figure S4.** Mean of the number of true leaves per seedling after exposure to ionising radiation. **Figure S5.** Comet assay - significant differences. **Figure S6.** Genotyping of the homozygous mutant plants. **Figure S7.** Bolting phenotype. **Table S1.** Primers to monitor gene expression (RT-PCR). **Table S2.** Relative variation of transcript steady-state content in Col, after the induction of DNA dsb damage. **Table S3.** Primers for qRT-PCR quantification of *AtRAD21* transcript steady state levels variation after exposure to ionising radiation. **Table S4.** Number of true leaves per seedling. **Table S5.** Mean, Mode and Median (true leaves per seedling). **Table S6.** Comet assay data. **Table S7.** Primers for mutants and complementation lines genotyping.

## Abbreviations

AEI: After end of irradiation exposure
C-NHEJ: Canonical non-homologous end-joining DNA repair pathway
Col: Wild-type Columbia-0
Comp: Complementation lines
dsb: Double strand break
GM: Germination solid medium
HR: Homologous recombination DNA repair pathway
NHEJ: Non-homologous end-joining DNA repair pathway
qRT-PCR: Quantitative real-time PCR
RT-PCR: Reverse transcription PCR
