# Supplementary material for: The AtRAD21.1 and AtRAD21.3 Arabidopsis cohesins play a synergistic role in somatic DNA double strand break damage repair
Source: BMC Plant Biol. 2014 Dec 16;14:353. doi: 10.1186/s12870-014-0353-9 (PMC4273318; doi:10.1186/s12870-014-0353-9)
Supplement: Additional file 1: — Materials and Methods Plant Material; qRT-PCR analysis; Comet assay. Figure S1. AtRAD21.1-GFP-6xHis transcript detection, and gene schematic representation. Figure S2. Relative variation of steady-state transcript levels during the 48 hours after the exposure to ionising radiation. Figure S3. Frequency of seedlings with different numbers of true leaves, in different genotypes, before and after exposure to ionising radiation. Figure S4. Mean of the number of true leaves per seedling after exposure to ionising radiation. Figure S5. Comet assay - significant differences. Figure S6. Genotyping of the homozygous mutant plants. Figure S7. Bolting phenotype. Table S1. Primers to monitor gene expression (RT-PCR). Table S2. Relative variation of transcript steady-state content in Col, after the induction of DNA dsb damage. Table S3. Primers for qRT-PCR quantification of AtRAD21 transcript steady state levels variation after exposure to ionising radiation. Table S4. Number of true leaves per seedling. Table S5. Mean, Mode and Median (true leaves per seedling). Table S6. Comet assay data. Table S7. Primers for mutants and complementation lines genotyping. [file 12870_2014_353_MOESM1_ESM.pdf]

Additional file
Materials and Methods, Tables and Figures

Title: The AtRAD21.1 and AtRAD21.3 Arabidopsis cohesins play a synergistic role in somatic DNA double strand break damage repair.

Authors: da Costa-Nunes JA, Capitão C, Kozak J, Costa-Nunes P, Ducasa GM, Pontes O, Angelis KJ

Additional file:
Materials and Methods

Plant material

Arabidopsis thaliana accession Columbia-0 (Col) was obtained from the Nottingham Arabidopsis Stock Centre. The accession Wassilewskija-1 (Ws) and the *atku80* mutant were kindly provided by Dr. C.E. West (Faculty of Biological Sciences, Leeds University, UK) [1]. The mutants *atrad21.1* (salk_044851), *atrad21.3* (salk_076116) and the double mutant *atrad21.1 atrad21.3* have been previously described [2]. The double mutant *atku80 atrad21.1* was obtained via controlled cross-pollination.

qRT-PCR analysis

Rosette leaves from non-irradiated and irradiated four weeks old Col plants grown in GM medium, were harvested and immediately frozen in liquid nitrogen, 5, 15, 30 and 45 minutes, and 1, 2, 4, 6, 8, 10, 24 and 48 hours after the 316 Gy irradiation sessions (2.65 Gy/minute; source: Co60). Three independent biological replicas (irradiated and non-irradiated) of four weeks old Col plants were obtained. Total RNA was extracted using the RNeasy Plant kit (Qiagen). The quality, quantity and integrity of all the RNA samples was assessed before being individually processed with the Turbo-DNA-free kit (Ambion). 1μg of total RNA from each individual sample was used for cDNA synthesis (using SuperScript III/RNaseOUT enzyme mix and Oligo(dt)$_{20}$).

qRT-PCR reaction was performed in the iQ™5 Real-Time PCR Detection System, in a 20µl reaction mix containing cDNA corresponding to 6.1ng of total RNA, 250nM of each primer and 10 µl of iQ™ SYBR Green Supermix (Bio-Rad). Quantification of gene expression was carried out after PCR amplification (1 cycle, 95°C for 3 minutes; 45 Cycles, 95°C for 10 seconds, 60°C for 10 seconds, 72°C for 10 seconds (with plate read); 1 Cycle, Melting curve from 55°C to 95.5°C, reading every 0.5°C, hold 10 seconds). Three different qRT-PCR reactions from each of the three cDNA replicas were carried out and quantified. For a given time point after the irradiation, the expression value attributed to each gene is the average from the three independent biological replicas. For each gene, the data was normalised using the expression level of non-irradiated samples as reference (arbitrary value of 1). *Actin2* [3] and *AtEF1αA4* [4] were used as reference genes. Relative quantification of transcript accumulation of the genes of interest, using *Actin2* and *AtEF1αA4* as reference genes, was obtained using the Pfaffl method [5]. Reference values for inter-plate calibration were obtained by amplifying, with the *Actin2* primers, a dilution series of a bulk mix of all cDNA samples. The quantification cycle (Cq) was determined using Bio-Rad iQ5 Optical System Software, Version 2.0, Standard Edition.

The primers (with a calculated Tm of approximately 60°C) were designed with the aid of the Netprimer program. Primers were empirically tested; the melting curve was analysed (iQ™5 Real-Time PCR) and the size and number of the PCR products assessed (not shown) in agarose gels. All the primer pairs promote PCR amplification of single PCR products with efficiency between 90% and 106%. Primer pairs sequence and additional information is provided here in, in Additional file Table S3. Each of the primers qRTmR21-1L, qRTR21-1R, qRTR21-2Rz, qRTR21-3Lx, qRTR21-3Rx, qRTR51-L and rtATM-R spans two adjacent exons. The primer qRTEF1-Lx spans the 5'UTR and the 1st exon. The two qRT-PCR amplicons from *AtRAD21.1* span the 9th and 10th exons (87bp), and the 12th and 13th exons (110bp). The amplicon from *AtRAD21.2* spans the 7th and 8th exons (108bp), and the one from *AtRAD21.3* spans the 12th, 13th and 14th exons (132bp). The amplicon from *AtRAD51* spans the 1st and 2nd exons, and that of the *AtATM* spans the 45th, 46th and 47th exons. Both the qRT-PCR amplicon

from *Actin2* and that from *AtEF1αA4* span the 5'UTRs and the 1st exon. The regions of the *AtRAD21* genes that are amplified (and the respective amplicon length) with the qRT-PCR primer pairs are depicted in Additional file Figure S1f).

Comet assay

Seeds were germinated under a 16 hours of light (at 22° C) followed by 8 hours of darkness (at 18° C) cycle, on Petri dishes containing ½MS media with Gamborg B5 Vitamins (Duchefa, Haarlem, The Netherlands) solidified with 0.8% Plant agar (Duchefa, Haarlem, The Netherlands) and overlaid with cellophane to facilitate collection of seedlings. Prior treatment, seedlings were gently transferred from cellophane to liquid ½MS media, to avoid dehydration.

Nuclear DNA fragmentation was measured in 10-days-old seedlings untreated and treated with 10 µg/ml or 30 µg/ml Bleomycin Sulfate (Bleomedac; Hamburg, Germany) for 1 hour in liquid ½MS. After Bleomycin treatment, seedlings were thoroughly rinsed in H_2O, blotted on filter paper and either immediately flash frozen in liquid nitrogen (t = 0) or left to recover in ½MS for the indicated repair times, before being frozen. DNA double strand breaks (dsb) were assayed using a neutral comet assay [6,7]. Plant material processing and data acquisition was carried out as described in previous reports [7]. In brief 70 µl of nuclear suspension obtained by chopping seedlings with a razor blade were dispersed in 280 µl of melted 0.7% LMT agarose (GibcoBRL, Gaithersburg, USA) and used to cast four gels on two microscopic slides per sample analysed. Comets were viewed in epifluorescence with a Nikon Eclipse 800 microscope after staining with SYBR Gold stain (Molecular Probes/Invitrogen, Eugene, USA) and evaluated by the Comet module of the LUCIA cytogenetics software suite (LIM, Praha, Czech Republic). The incidence of DNA dsb damage was measured as the fraction of fragmented DNA that moved from the comet head to the comet tail (% tail-DNA). The calculated percentage of damage remaining for each given repair time t_X is defined as:

$$K(tx) = \%\text{ damage remaining}(tx) = \frac{\text{mean }\%\text{tail-DNA}(tx) \;-\; \text{mean }\%\text{tail-DNA}(\text{control})}{\text{mean }\%\text{tail-DNA}(t0) \;-\; \text{mean }\%\text{tail-DNA}(\text{control})} \text{x } 100$$

Data for Arabidopsis Col and the mutant lines (*atrad21.1*, *atrad21.3*, *atrad21.1 atrad21.3*) analysed in this study were measured in three independent

experiments and compiled. DNA dsb in these Arabidopsis mutants and Col were measured as the % tail-DNA in control seedlings and all mutated lines without a treatment, and in the following seven time-points: 0, 3, 5, 10, 20, 60 and 180 minutes after the treatment. 25 evaluated comets per independent gel replica, total in, at least, 300 comets analysed per experimental point. Microscopic slides were coded, and blind measured and evaluated.

1. West CE, Waterworth WM, Story GW, Sunderland PA, Jiang Q, Bray CM: **Disruption of the Arabidopsis *AtKu80* gene demonstrates an essential role for AtKu80 protein in efficient repair of DNA double-strand breaks *in vivo*.** *Plant J* 2002, **31**(4):517-528.
2. da Costa-Nunes JA, Bhatt AM, O'Shea S, West CE, Bray CM, Grossniklaus U, Dickinson HG: **Characterization of the three *Arabidopsis thaliana RAD21* cohesins reveals differential responses to ionizing radiation.** *J Exp Bot* 2006, **57**(4):971-983.
3. Watanabe K, Pacher M, Dukowic S, Schubert V, Puchta H, Schubert I: **The STRUCTURAL MAINTENANCE OF CHROMOSOMES 5/6 complex promotes sister chromatid alignment and homologous recombination after DNA Damage in *Arabidopsis thaliana*.** *Plant Cell* 2009, **21**(9):2688-2699.
4. Endo M, Ishikawa Y, Osakabe, Nakayama S, Kaya H, Araki T, Shibahara K, Abe K, Ichikawa H, Valentine L, Hohn B, Toki S: **Increased frequency of homologous recombination and T-DNA integration in Arabidopsis CAF-1 mutants.** *EMBO J* 2006, **25**(23):5579-5590.
5. Pfaffl MW: **A new mathematical model for relative quantification in real-time RT-PCR.** *Nucleic Acids Res* 2001, **29**(9):e45.
6. Olive PL, Banáth JP: **The comet assay: a method to measure DNA damage in individual cells.** *Nature Protocols* 2006, **1**(1):23-29.
7. Kozak J, West CE, White C, da Costa-Nunes JA, Angelis KJ: **Rapid repair of DNA double strand breaks in *Arabidopsis thaliana* is dependent on proteins involved in chromosome structure maintenance.** *DNA Repair* 2009, **8**(3):413-419.

Additional file:

Figure S1

AtRAD21.1-GFP-6x_His_ transcript detection, and gene schematic representation.

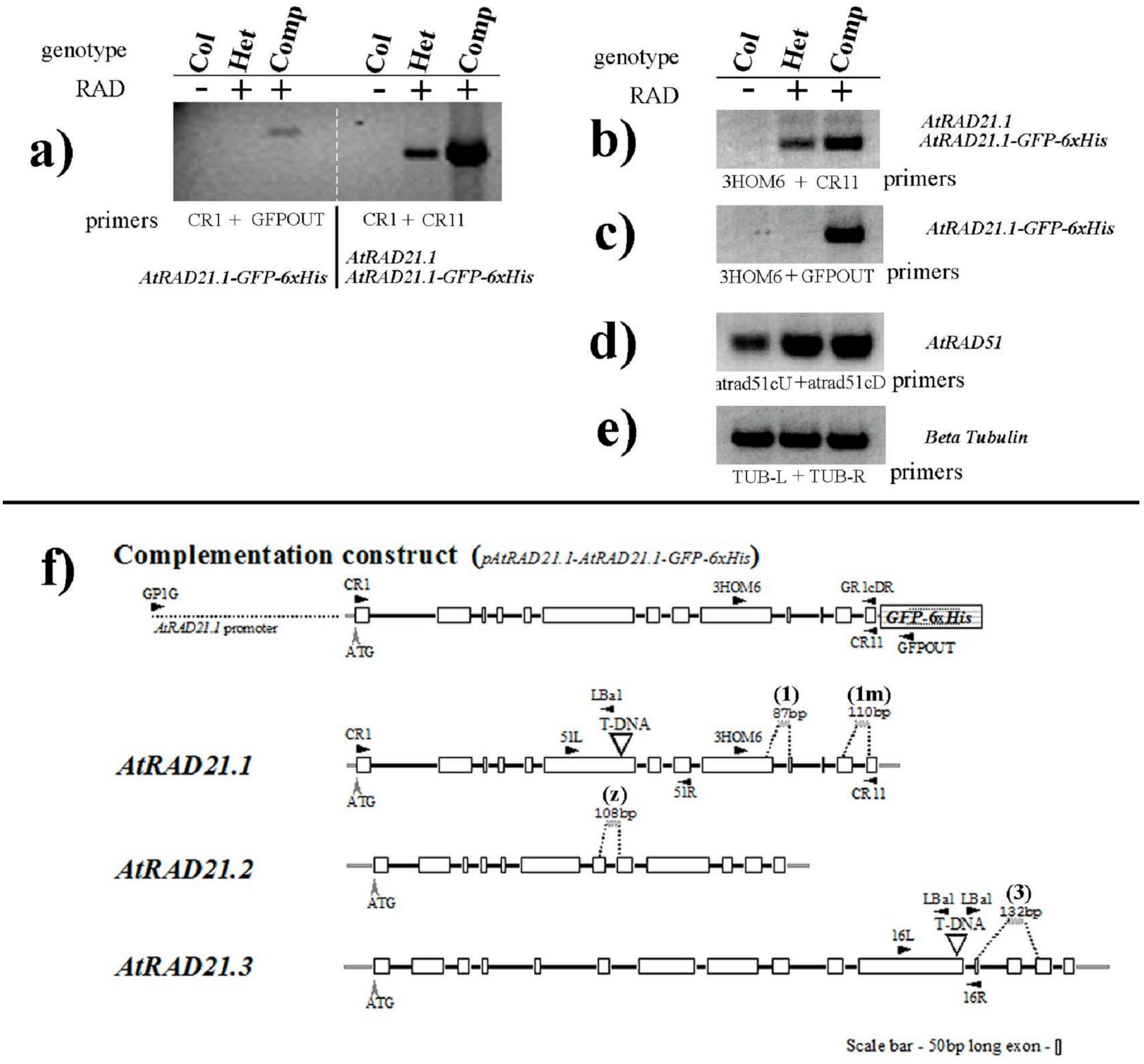

Figure S1 legend:

The complementation line (Comp) expresses the *AtRAD21.1-GFP*-6x*His* transcript.

AtRAD21.1-GFP-6x*His* gene expression is unequivocally detected in the complementation line (Comp); **a)** (primer pair CR1 and GFPOUT); **c)** (primer pair 3HOM6 and GFPOUT). High levels of *AtRAD21.1* and *AtRAD21.1-GFP*-6x*His* gene expression (**a**, **b**, **c**) are detected in samples (Het and Comp) exposed to ionising radiation (+) (a DNA dsb inducing agent). The higher levels of expression of the *AtRAD51* gene in the Het and Comp samples confirm that these samples were exposed to ionising radiation (+) (**d**) [1]. *Beta Tubulin* gene family expression data was used as control, showing that the detected differences in gene expression levels are not due to differences in cDNA template input (**e**) [2]. Samples non-exposed to DNA dsb inducing agents (Col -) have low/undetectable *AtRAD21.1* transcript content [3].

Genotypes:

Col - wild-type Columbia-0 plant

Het - *AtRAD21.1 atrad21.1* heterozygous plant

Comp - *atrad21.1* homozygous mutant containing the complementation construct.

RAD: (-) non-irradiated sample; (+) samples harvested 1 hour after the exposure to ionising radiation (150 Gy; 3.25 Gy/minutes; source: Cs137).

Comp and Het were irradiated to increase the *AtRAD21.1* (and expecting to increase *AtRAD21.1-GFP-6xHis*) transcript content, to facilitate the detection of the transcript by RT-PCR.

Samples Col and Het were harvested from rosette leaves; samples from Comp were harvested from seedlings. According to previous reports [3], both tissues (seedlings and rosette leaves) have undetectable or low transcript levels of *AtRAD21.1* transcript in non-irradiated samples, but both accumulate *AtRAD21.1* transcripts after exposure to ionising radiation. In contrast, the *atrad21.1* mutant allele does not exhibit this increase in gene expression after exposure to ionising radiation.

PCR products (**a** to **e**) were amplified with the primers: **a)**: CR1, GFPOUT and CR1, CR11; **b)**: 3HOM6, CR11; **c)**: 3HOM6, GFPOUT; **d)**: atrad51cU, atrad51cD; **e)**: TUB-L, TUB-R.

The primer pairs CR1 and CR11, and 3HOM6 and CR11, amplify the cDNA from both the native *AtRAD21.1* transcript and the *AtRAD21.1*-*GFP*-6x*His* transcript (**a**, **b**). The PCR products obtained with the GFPOUT and CR1, and GFPOUT and 3HOM6 primer pairs amplify solely the complementation construct's cDNA (**a**, **c**); these primer pairs do not amplify spurious products from the *AtRAD21.1* wild-type allele (in Col and Het) nor from the *atrad21.1* alleles (in heterozygous (Het) *AtRAD21.1 atrad21.1*) in plants exposed and non-exposed to γ-rays. Primer pair atrad51cU and atrad51cD attests which samples were non-exposed (low expression) and exposed (high expression) to DNA dsb ionising radiation (**d**). TUB-L - TUB-R PCR products were used to certify that the cDNA input was identical in all PCR reactions (**e**).

Vertical line (in image **A**) separates two different PCR products photographed in the same gel.

(f) Schematic representation of the *pAtRAD21.1-AtRAD21.1-GFP-6xHis* complementation construct and the *AtRAD21* genes, depicting primers position (black triangles), gene structure (adapted from [3]), the qRT-PCR amplicons (light grey rectangles), the amplicons' length (87, 110, 108, 132bp), and their respective names (**1**, **1m**, **z**, **3**).

Exons (open boxes), introns (black lines), and 3' and 5'UTR of the three *Arabidopsis RAD21* paralogs (grey lines). Grey arrowhead (ATG codon); Inverted open triangle (T-DNA). The grey rectangles (qRT-PCR amplicons) span over more then one exon.

The upstream genomic sequence of *AtRAD21.1* (*AtRAD21.1* promoter; *pAtRAD21.1*) is represented by the dotted horizontal line; this is not represented in the same scale used for the *AtRAD21* genes (scale bar: 50bp).

1. Klimyuk VI, Jones JDG: ***AtDMC1*, the Arabidopsis homologue of yeast *DMC1* gene: characterization, transposon-induced allelic variation and meiosis-associated expression.** *Plant J* 1997, **11**(1):1-14.

2. Knight H, Veale E, Warren GJ, Knight MR: **The *sfr6* mutation in *Arabidopsis* suppresses low-temperature induction of genes dependent on the CRT/DRE sequence motif.** *Plant Cell* 1999, **11**(5):875-886.

3. da Costa-Nunes JA, Bhatt AM, O'Shea S, West CE, Bray CM, Grossniklaus U, Dickinson HG: **Characterization of the three *Arabidopsis thaliana RAD21* cohesins reveals differential responses to ionizing radiation.** *J Exp Bot* 2006, **57**(4):971-983.

Additional file:

Figure S2

Relative variation of steady-state transcript levels during the 48 hours after the exposure to ionising radiation

(A)

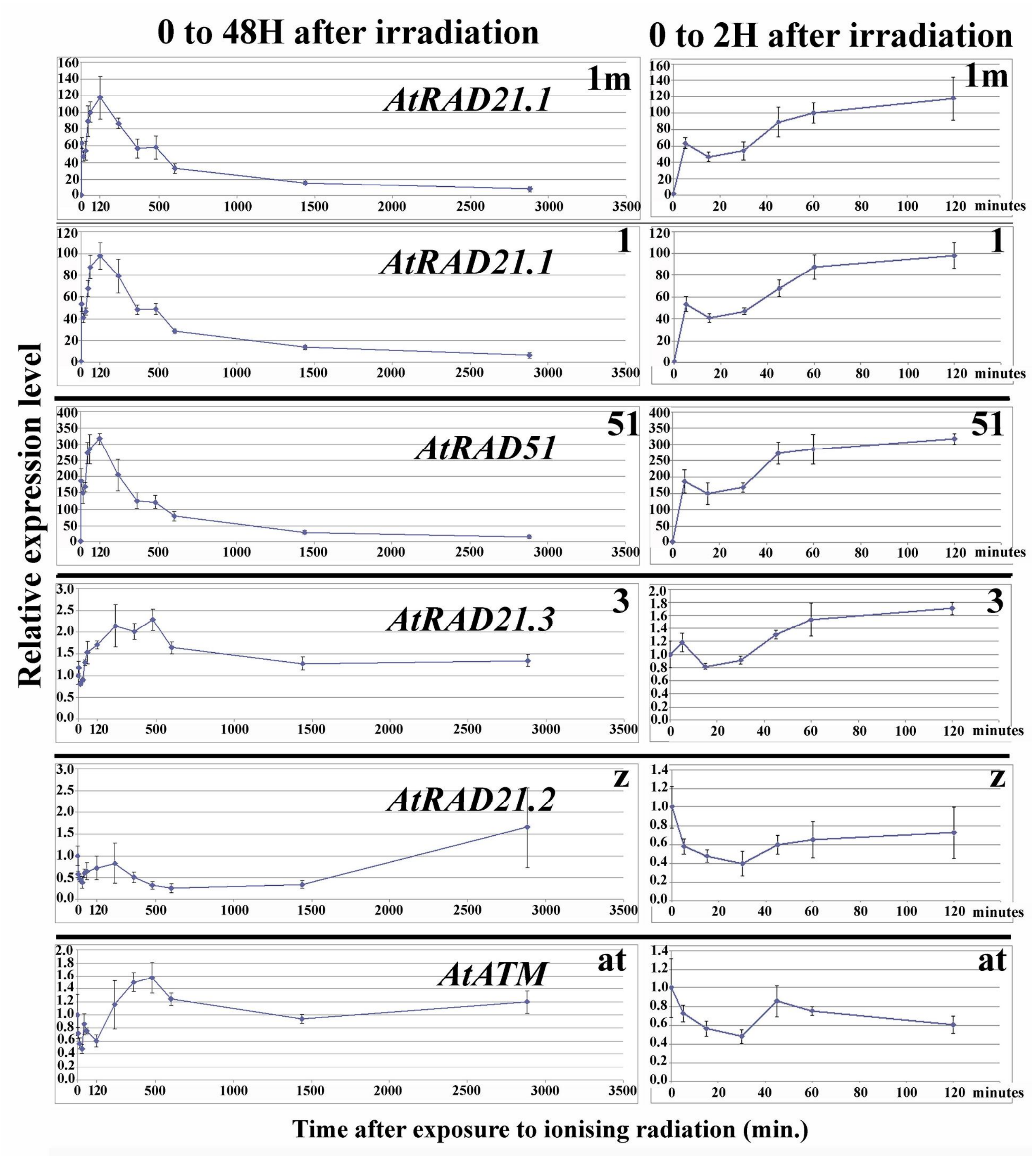

Figure S2

(B)

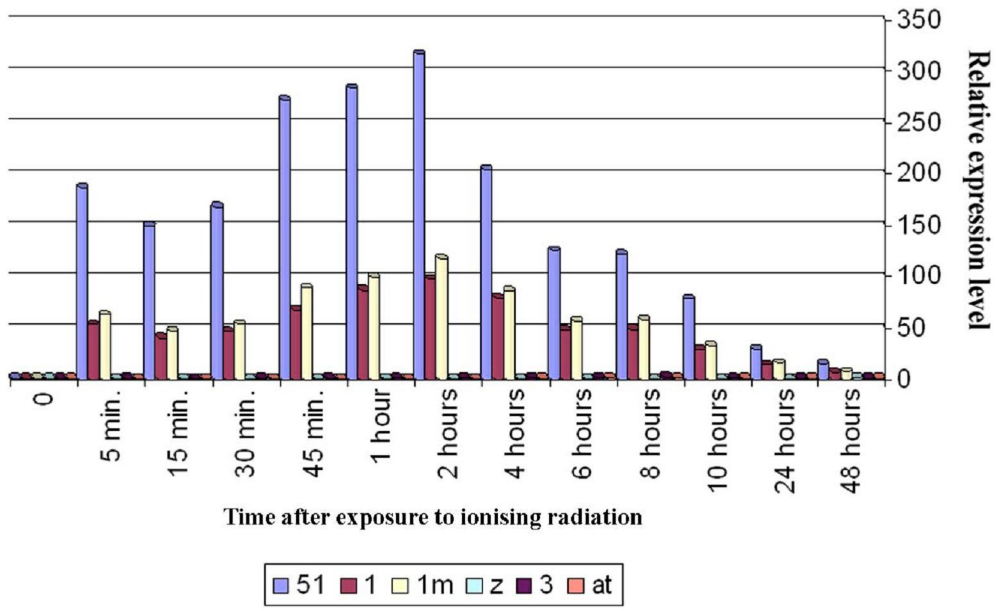

Figure S2 legend:

(A) - Relative variation of steady-state transcript levels, in four weeks old Col rosette leaves irradiated with 316 Gy (2.65 Gy/minute; source: Co60), during the 48 hours (2880 minutes) (left column), and detail of only the first 2 hours (120 minutes) (right column), after the exposure to ionising radiation. A sharp increase in *AtRAD21.1* transcript steady state levels is detected as early as 5 minutes AEI (right column); data obtained using two different primer pairs that amplify two different amplicons (**1** and **1m**). *AtRAD21.1* and *AtRAD51* (**51**) steady-state transcript level variation patterns are similar (left column). In comparison, relative variation in *AtRAD21.2* (**z**), *AtRAD21.3* (**3**) and *AtATM* (**at**) steady-state transcript level is not as dramatic. Error bars represent the standard deviation.

(B) - Compiled data from the six charts shown in **Fig. S2 (A)**, to illustrate the broad difference in relative transcript content variation, detected by qRT-PCR, of the *AtRAD21.1* gene (**1** and **1m**) and *AtRAD51* (**51**), in contrast with that of

AtRAD21.2 (**z**), *AtRAD21.3* (**3**) and *AtATM* (**at**), during the first 48 hours after exposure to ionising radiation. The steady-state transcript levels of non-irradiated samples was used as reference (with the arbitrary value: 1). **0**- non-irradiated samples.

Samples (rosette leaves) were harvested **5**, **15**, **30**, and **45** minutes, and 60, 120, 240, 360, 480, 600, 1440 and 2880 minutes (i.e. **1**, **2**, **4**, **6**, **8**, **10**, **24** and **48** hours, respectively) after the end of irradiation.

Sequence of the primer pairs amplifying the qRT-PCR amplicons (**51**, **1**, **1m**, **z**, **3** and **at**) is provided in Additional file Table S3, and qRT-PCR data is provided in Additional file Table S2.

Col - wild-type Columbia-0

min. -minutes

Additional file:

Figure S3

Frequency of seedlings with different numbers of true leaves, in different genotypes, before and after exposure to ionising radiation

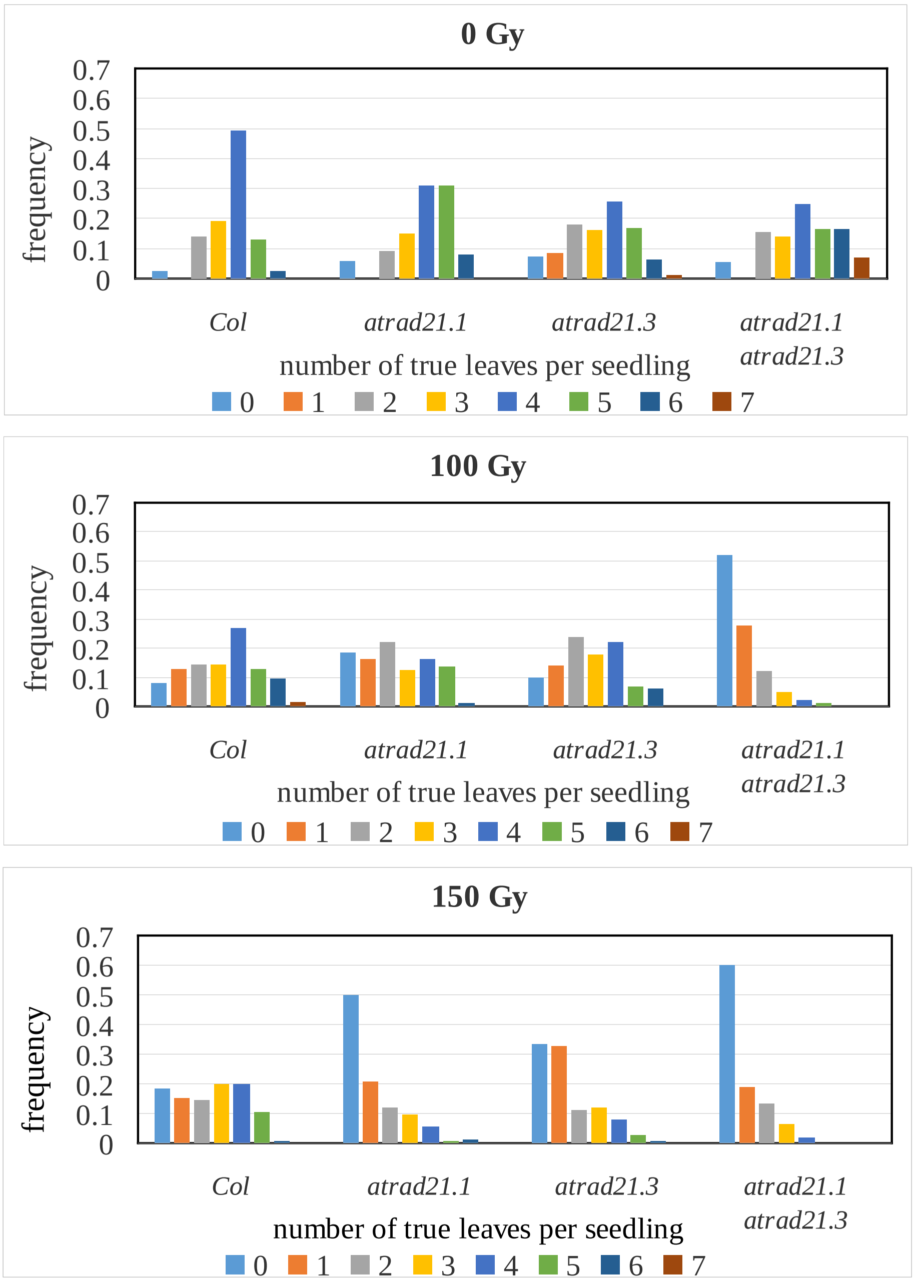

x axis: genotypes and the number of true leaves per seedling (from 0 to 7).

y axis: frequency of seedlings with 0, 1, 2, 3, 4, 5, 6 or 7 true leaves; the sum of frequencies equals 1.

Figure S3 legend:

The *atrad21.1 atrad21.3* double mutant is the *atrad21* mutant with the highest hypersensitivity to ionising radiation (as assessed via the number of true leaves per seedling). The *atrad21.1 atrad21.3* double mutant requires a lower dose of ionising radiation (100 Gy) to exhibit a higher frequency of seedlings with none or one true leaf. In contrast, under the experimental conditions used, the *atrad21.1* and *atrad21.3* single mutants only exhibit a high frequency of seedlings with few true leaves (0 and 1) when exposed to higher doses of ionising radiation (γ-rays; 150 Gy). At 150 Gy, the *atrad21.1* mutant has a higher incidence of seedlings with 0 and 1 true leaves than the *atrad21.3* mutant.

The total number of seedlings used in the calculation of the frequencies was the following at 0 Gy: Col (79), *atrad21.1* (87), *atrad21.3* (94), *atrad21.1 atrad21.3* (72). At 100 Gy: Col (63), *atrad21.1* (87), *atrad21.3* (101), *atrad21.1 atrad21.3* (83). At 150 Gy: Col (124), *atrad21.1* (158), *atrad21.3* (117), *atrad21.1 atrad21.3* (112). The total number of seedlings is shown between brackets. 0 Gy - non-exposed to ionising radiation. 100 Gy, 150 Gy - exposed to 100 Gy (or 150 Gy) of ionising radiation (γ-rays; 3.25 Gy/minute; source: Cs137). Col - Wild type Col-0

Additional file:

Figure S4

Mean of the number of true leaves per seedling after exposure to ionising radiation

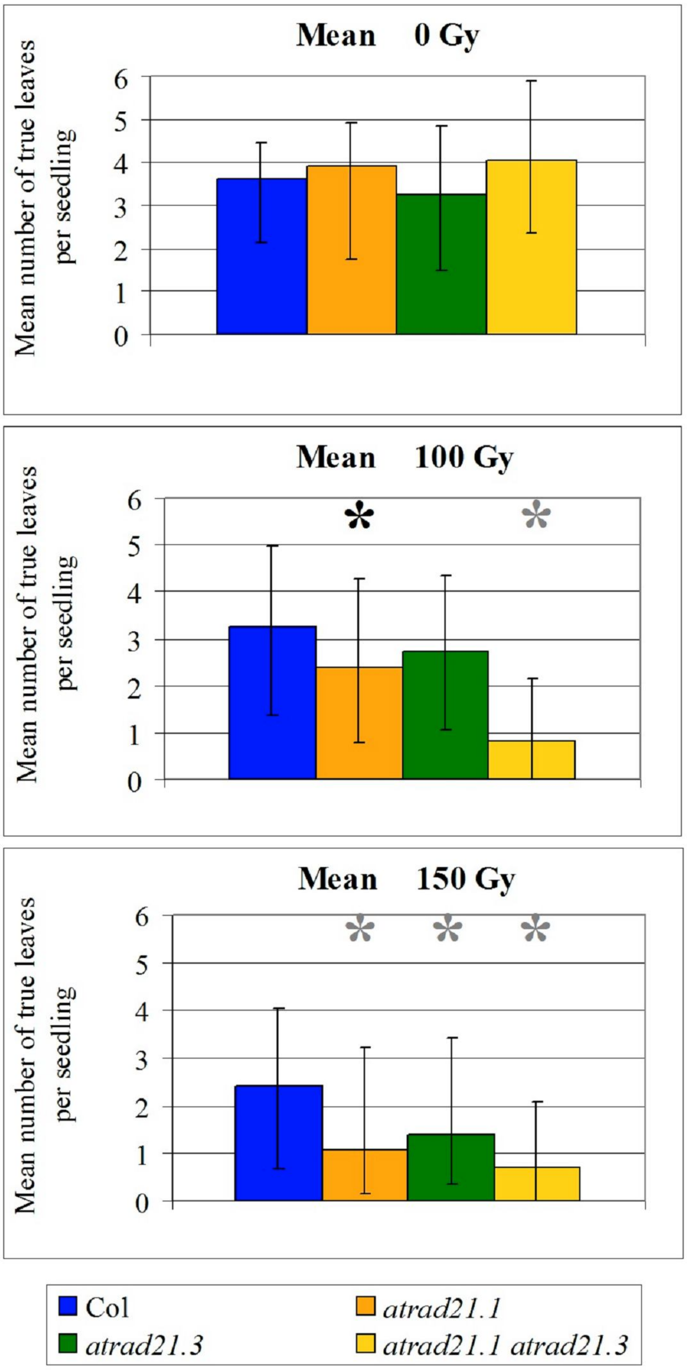

Figure S4 legend:

Both the median (Figure 4) and the mean (Additional files Figure S4 and Table S5) illustrate the dramatic increase in the number of seedlings which either do not have any true leaf, or only have one or two true leaves per seedling, 15 days after the exposure to radiation. The mean illustrates less clearly the differences between the wild type and the *atrad21* mutants, most likely due to the effect of outliner values that distort the value of the mean in skewed populations (in particular in the *atrad21.1 atrad21.3* double mutant at 100 Gy and 150 Gy, and in the *atrad21* mutant at 150 Gy).

The reduced number of true leaves per seedling, that is characteristic to plants exposed to DNA damage inducing ionising radiation, is illustrated in Figure 3 as well as in other sources [1,2]. Error bars represent the standard deviation of the data above and below the mean. Black asterisk denotes significant difference between wild type Columbia-0 (Col) and *atrad21.1*, 15 days after exposure to 100 Gy (Mann-Whitney U=2026; p value (p)=0.00652). Grey asterisk denotes significant difference (p=0) between the *atrad21.1 atrad21.3* mutant and Col at 100 Gy (Mann-Whitney U=726.5; p=0), and between *atrad21* mutants and Col 15 days after exposure to 150 Gy (Col *versus atrad21.1*; Mann-Whitney U=5278.5; p=0), (Col *versus atrad21.3*; Mann-Whitney U=4712; p=0), (Col *versus atrad21.1 atrad21.3*; Mann-Whitney U=2920.5; p=0). At 0 Gy, none of the *atrad21* single and double mutants is significantly different from Col (Col *versus atrad21.1*; Mann-Whitney U=2769; p=0.03078), (Col *versus atrad21.3*; Mann-Whitney U=3297; p=0.20408), (Col *versus atrad21.1 atrad21.3*; Mann-Whitney U=2346.5; p=0.06432). Statistical analysis was carried out using the Mann-Whitney non-parametric U-test ($p<0.01$, 2-tailed hypothesis).

The total number of seedlings used in the calculation of the means was the following at 0 Gy: Col (79), *atrad21.1* (87), *atrad21.3* (94), *atrad21.1 atrad21.3* (72). At 100 Gy: Col (63), *atrad21.1* (87), *atrad21.3* (101), *atrad21.1 atrad21.3* (83). At 150 Gy: Col (124), *atrad21.1* (158), *atrad21.3* (117), *atrad21.1 atrad21.3* (112). The total number of seedlings is shown between brackets. 0 Gy - non-exposed to ionising radiation. 100 Gy, 150 Gy - exposed to 100 Gy (or 150 Gy) of ionising radiation (γ-rays; 3.25 Gy/minute; source: Cs137). Col - Wild type Col-0

1. Friesner J, Britt AB: ***Ku80*- and *DNA ligase IV*-deficient plants are sensitive to ionizing radiation and defective in T-DNA integration.** *Plant J* 2003, **34**(4):427-440.
2. da Costa-Nunes JA, Bhatt AM, O'Shea S, West CE, Bray CM, Grossniklaus U, Dickinson HG: **Characterization of the three *Arabidopsis thaliana RAD21* cohesins reveals differential responses to ionizing radiation.** *J Exp Bot* 2006, **57**(4):971-983.

Additional file:

Figure S5

Comet assay – significant differences

DNA dsb damage induction and repair

(after DNA dsb damage induction with 30µg Bleomycin)

% of damaged (dsb) DNA in *nuclei* of *atrad21* mutants and Col

after induction of DNA dsb (0 to 180 minutes)

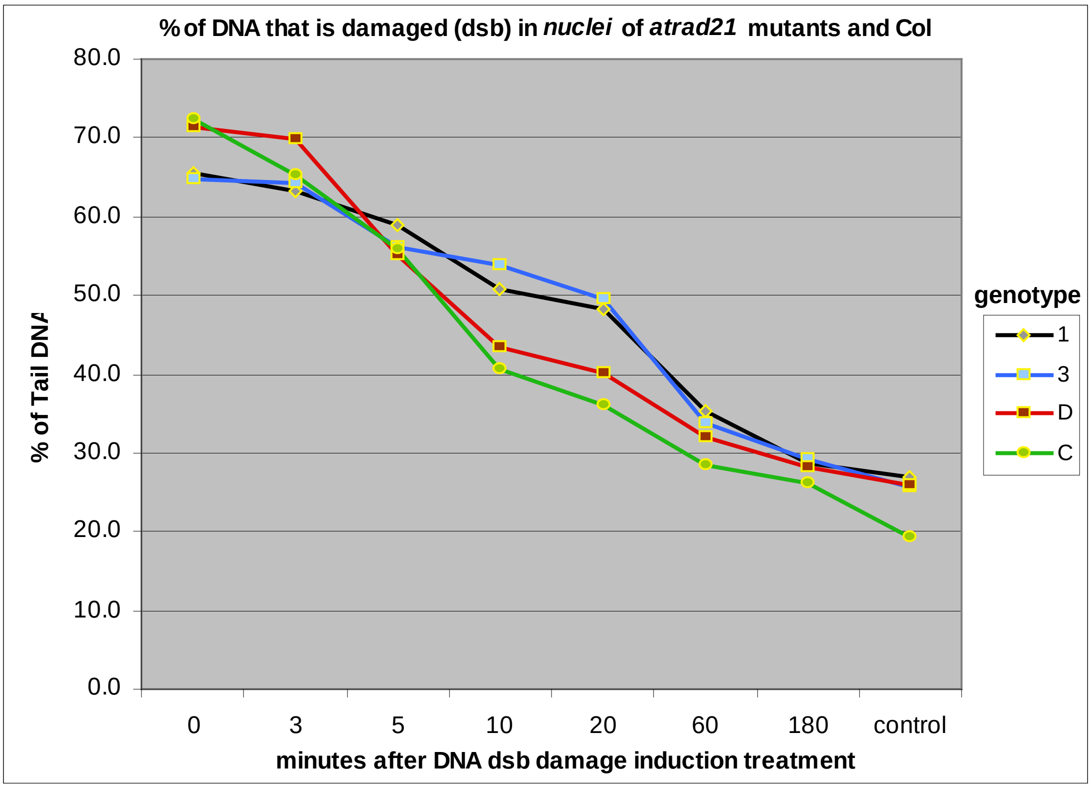

Graphics illustrating the significant differences in nuclear DNA dsb fragments content between two different genotypes, at 10, 20 and 60 minutes after the induction of DNA dsb damage

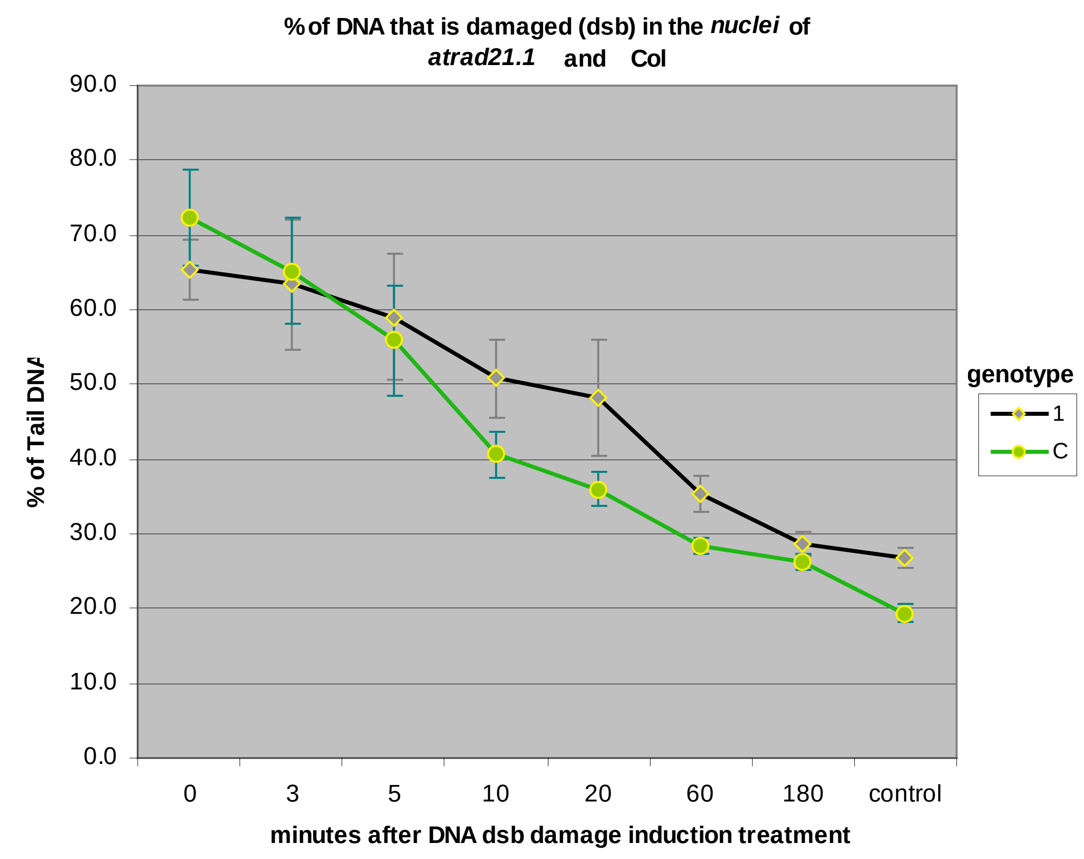

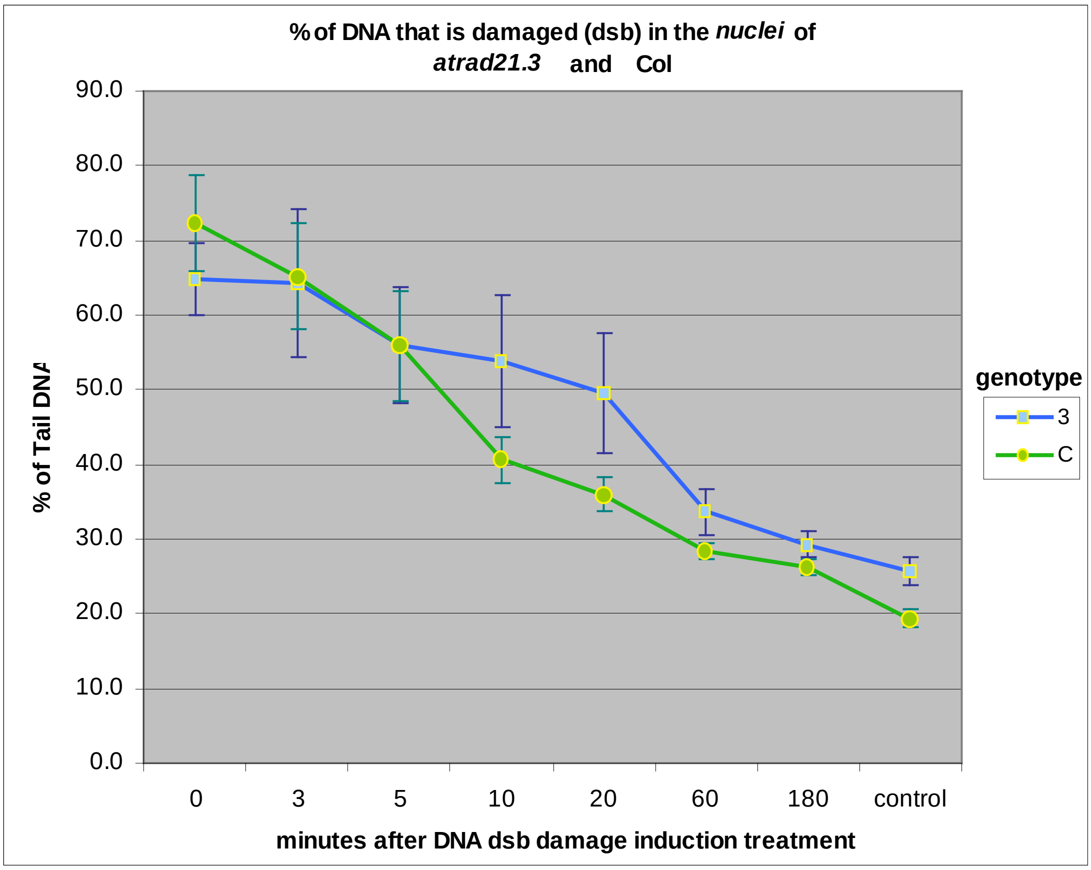

Graphic illustrating the significant differences in nuclear DNA dsb fragments

content between two different genotypes,

at 10, 20 and 60 minutes after the induction of DNA dsb damage

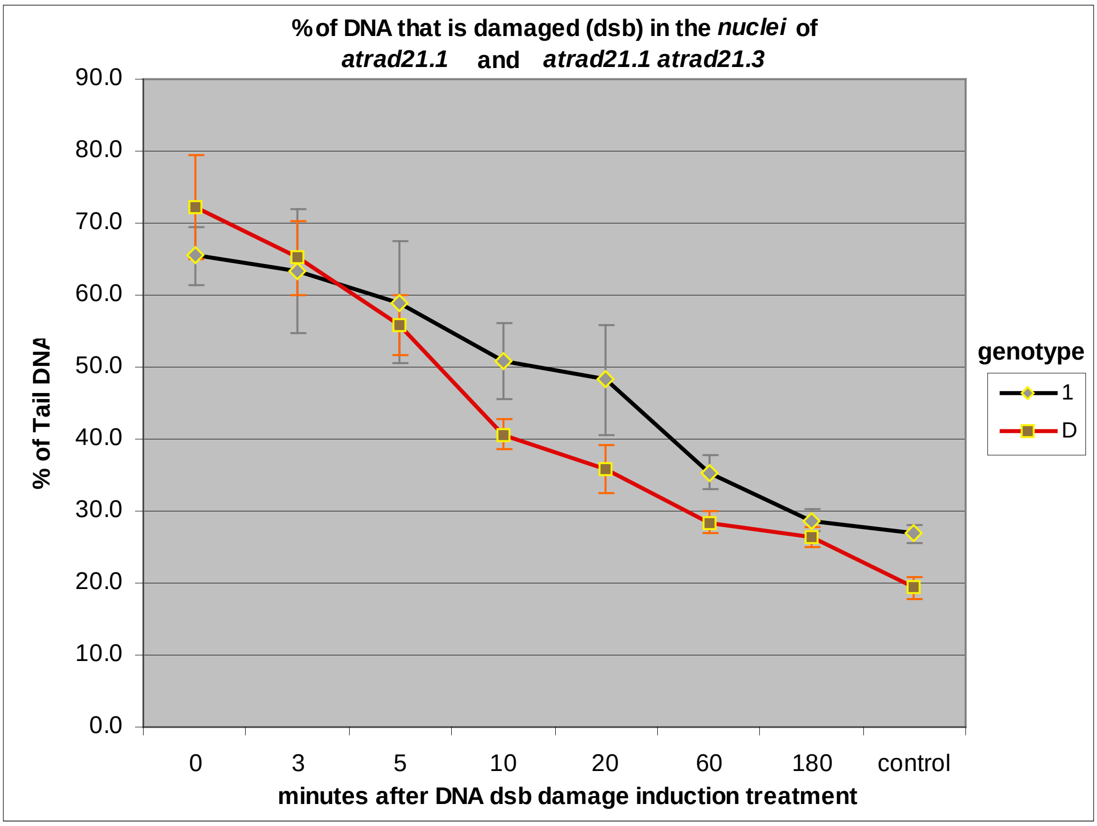

Graphics illustrating no significant differences in nuclear DNA dsb fragments content between two different genotypes

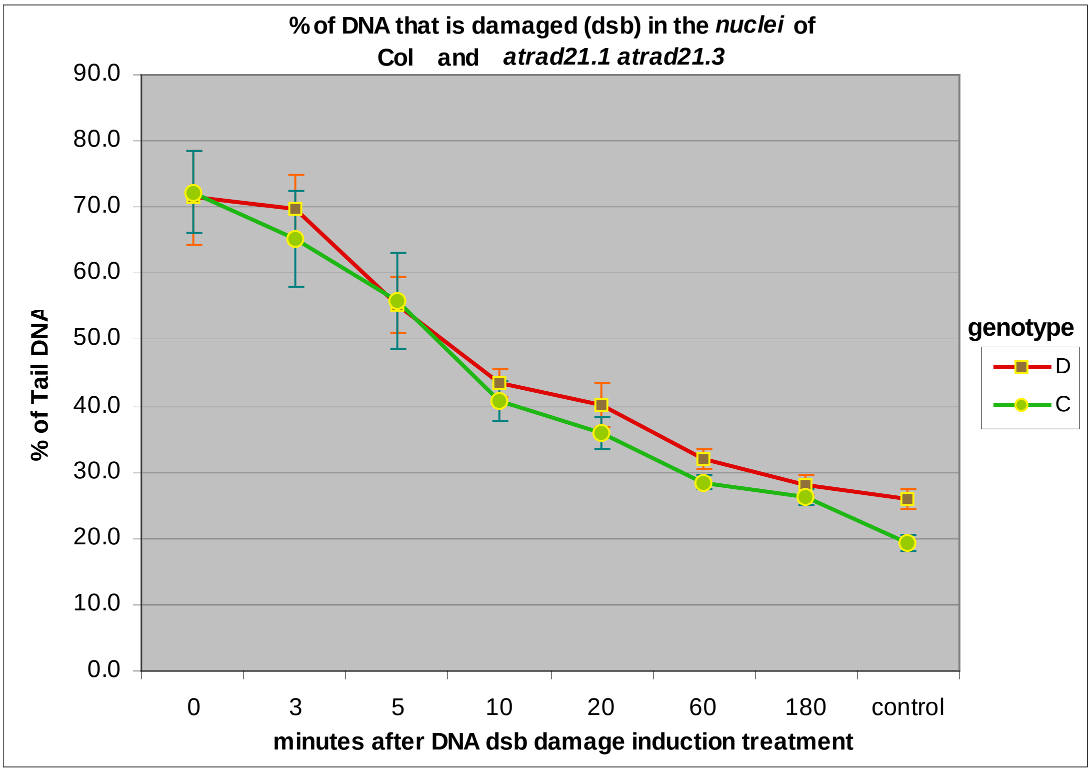

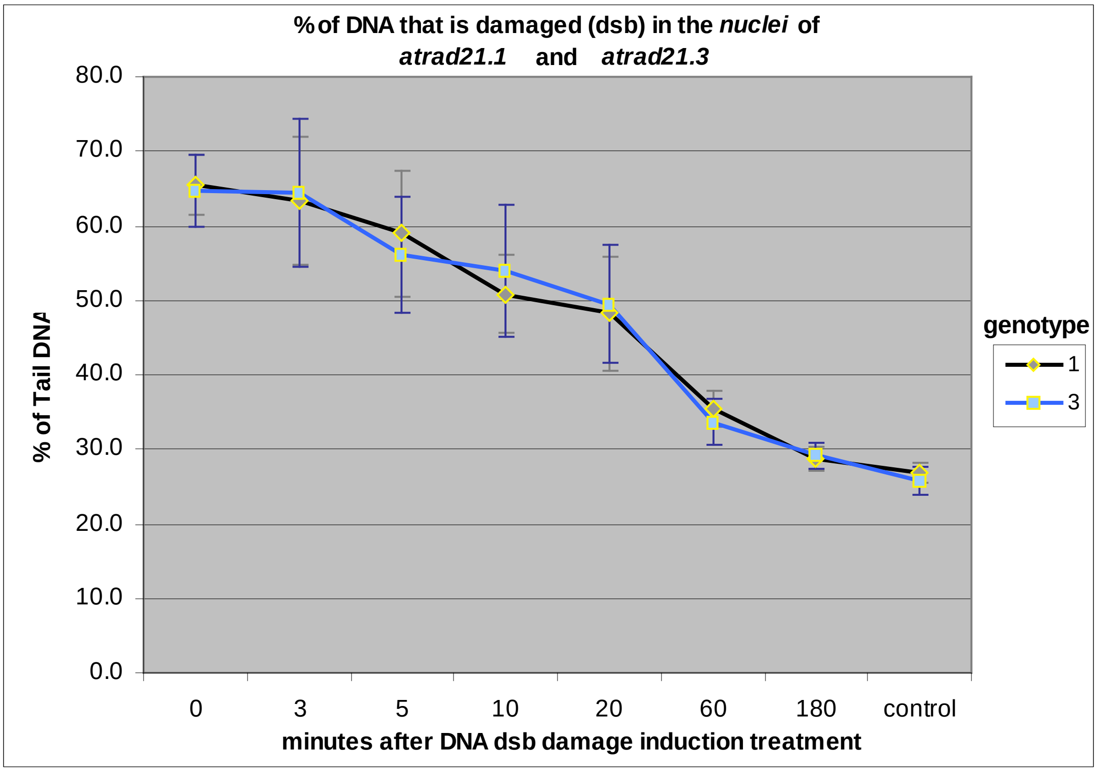

Graphic illustrating no significant differences in nuclear DNA dsb fragments content between two different genotypes

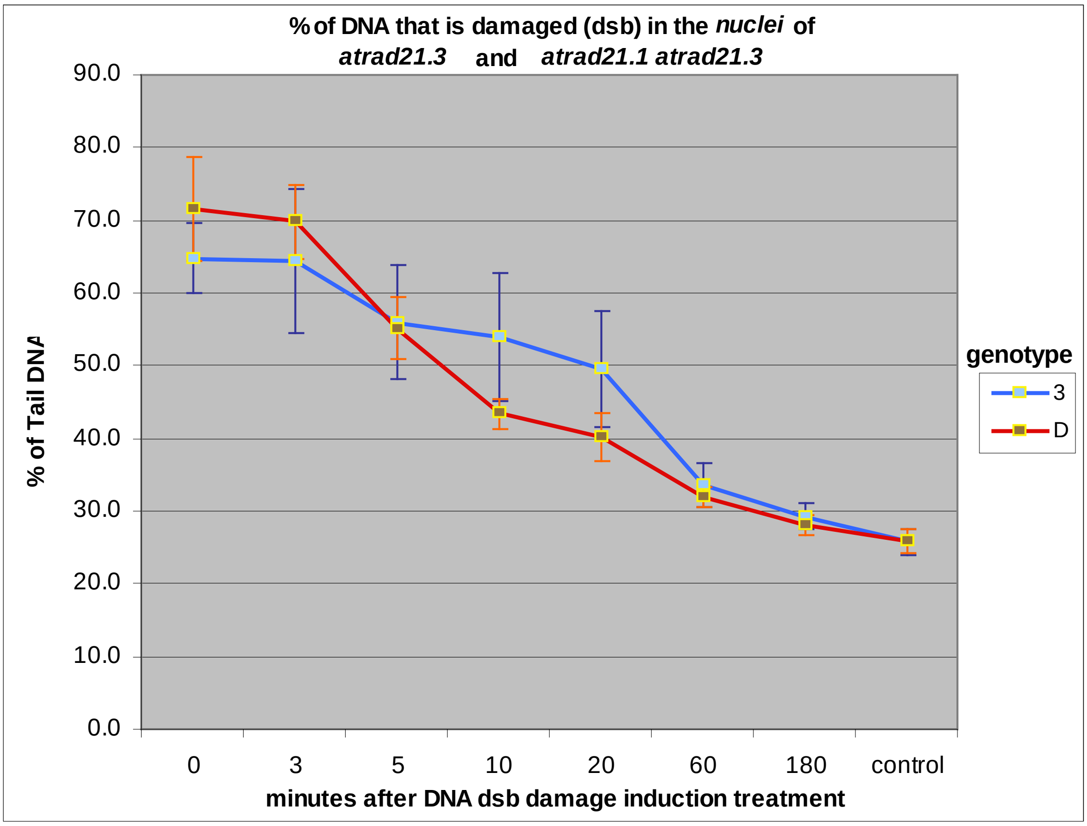

Figure S5 legend:

dsb - double strand break

% Tail DNA - % of dsb damaged DNA in the nucleus ; (Y-axis)

error bars represent the standard error (SE)

Genotype:

1 - *atrad21.1* homozygous mutant

3 - *atrad21.3* homozygous mutant

D - *atrad21.1 atrad21.3* double homozygous mutant

C - Col ; wild-type Columbia-0

Data (% Tail DNA; SE) is provided in Additional file Table S6.

Additional file:

Figure S6

Genotyping of the homozygous mutant plants

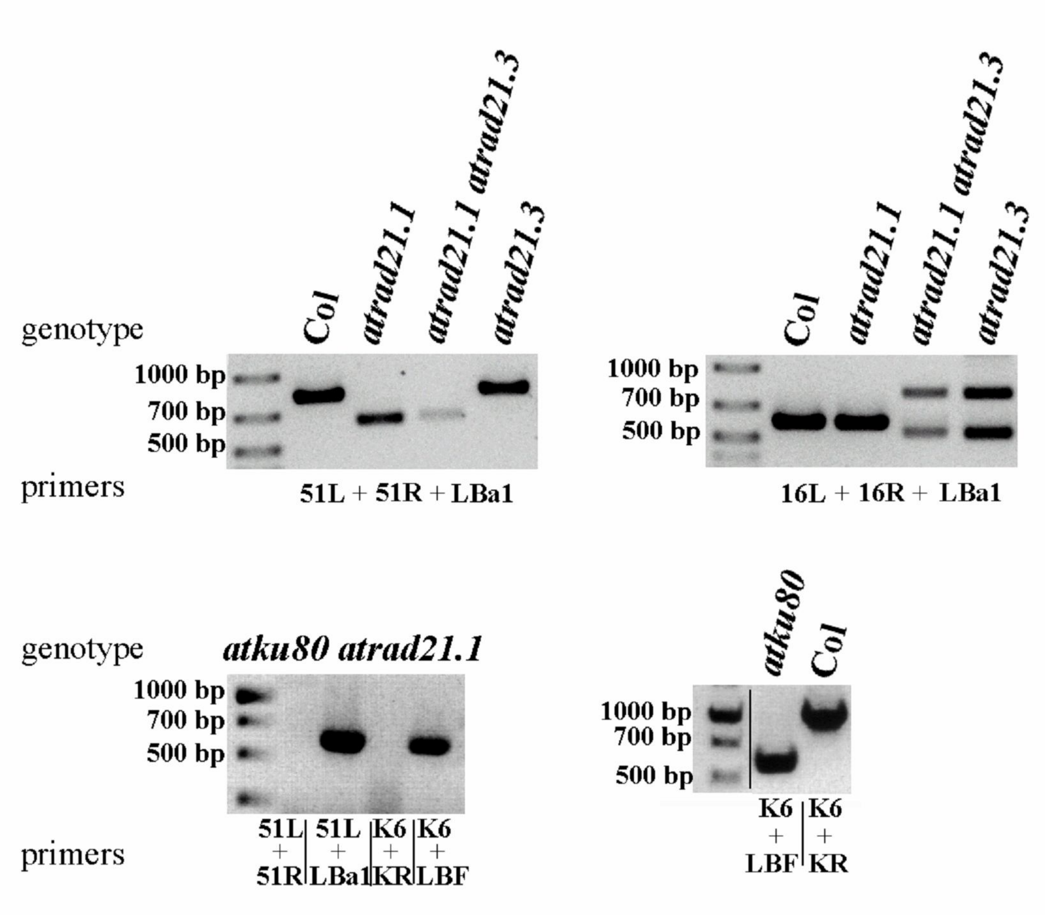

Figure S6 legend:

Top row:

Genotyping of Col, and *atrad21.1*, *atrad21.1 atrad21.3* and *atrad21.3* mutants using the primer mix (51L+ 51R + LBa1) and the primer mix (16L + 16 R + LBa1).

The amplicon (*AtRAD21.1* allele specific) amplified with the primers 51L and 51R has a higher molecular weight than the amplicon (*atrad21.1* allele specific) amplified with the 51L and LBa1 primers.

Only one *AtRAD21.3* allele specific amplicon (16L and 16R) is amplified with the primer mix 16L, 16R and LBa1. The combination of the three primers 16L, 16R and LBa1 (that targets the *atrad21.3* allele) yields two amplicons with different molecular weights.

Schematic drawing of gene structure (*AtRAD21.1* and *AtRAD21.3*) and primers relative position is depicted in Additional file Figure S1f).

Bottom row:

Genotyping of *atku80 atrad21.1* double homozygous mutant, *atku80* single homozygous mutant and Col using the primer pairs 51L and 51R (that target the *AtRAD21.1* allele), 51L and LBa1 (that target the *atrad21.1* allele), K6 and KR (that target the *AtKu80* allele) and K6 and LBFel (LBF) (that target the *atku80* allele).

Black vertical line in the *atku80* and Col gel separates two sections of the same agarose gel.

Primers sequence is provided in Additional file Table S7.

Col - wild-type Columbia-0

Additional file:

Figure S7

Bolting phenotype

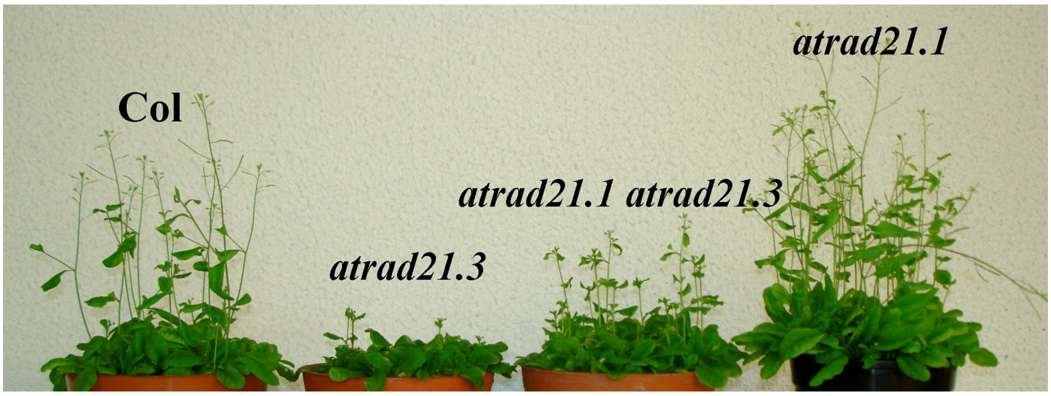

Figure S7 legend:

Bolting phenotytpe of the *atrad21* mutants. Vegetative growth of six weeks old *atrad21* mutants and Col plants is comparable. While bolting time in *atrad21.3* is delayed, and in *atrad21.1* it is wild type-like (Col) [1], the *atrad21.1 atrad21.3* double mutant exhibits an intermediate phenotype. This observation was carried out several times, with all four genotypes being sown and grown simultaneously in soil (in the same substract), in different growth chambers. These plants were not exposed to ionising radiation. Col - wild-type Columbia-0

1. da Costa-Nunes JA, Bhatt AM, O'Shea S, West CE, Bray CM, Grossniklaus U, Dickinson HG: **Characterization of the three *Arabidopsis thaliana RAD21* cohesins reveals differential responses to ionizing radiation.** *J Exp Bot* 2006, **57**(4):971-983.

Additional file:

Table S1

Primers to monitor gene expression (RT-PCR)

Gene	Primer	Primer sequence	PCR conditions
AtRAD21.1 <u>*At5g40840*</u>	CR1 [1]	ATGTTTTACTCGCATTGTCTAG	Annealing: 55°C, 45'' extention: 72°C, 3' 37 extention cycles amplifies wild type allele and Comp.
	CR11 [1]	CAAGCTTTTTGTGGTCTGGA	
AtRAD21.1	CR1 [1]	See above	Annealing: 55°C, 45'' extention: 72°C, 3' 37 extention cycles amplifies Comp. only
	GFPOUT +	GTATGTTGCATCACCTTCAC	
AtRAD21.1	3HOM6 +	GTAACGTGGTTTCGGTTGAG	Annealing: 54°C, 45'' extention: 72°C, 1' 35 extention cycles amplifies wild type allele and Comp.
	CR11 [1]	See above	
AtRAD21.1	3HOM6 +	See above	Annealing: 56°C, 45'' extention: 72°C, 1' 35 extention cycles amplifies Comp. only
	GFPOUT +	See above	
AtRAD51 <u>*At5g20850*</u>	Atrad51 cU +	AGCCATGATATTCCCACCAATC	Annealing: 54°C, 45'' extention: 72°C, 1' 35 extention cycles amplifies wild type allele
	Atrad51 cD +	GACTTGTCAACTTCCCATGG	

β-tubulin	TUB-L [2]	CCTGATAACTTCGTCTTTGG	Annealing: 60°C, 45’’<br>Extention: 72°C, 1.5’<br>25 extention cycles
gene family	TUB-R [2]	GTGAACTCCATCTCGTCCAT	amplifies wild type allele

+ primers designed for this study

Comp - complementation construct: gDNA *pARAD21.1-AtRAD21.1-GFP*-6x*His*

1. da Costa-Nunes JA, Bhatt AM, O’Shea S, West CE, Bray CM, Grossniklaus U, Dickinson HG: **Characterization of the three *Arabidopsis thaliana RAD21* cohesins reveals differential responses to ionizing radiation.** *J Exp Bot* 2006, **57**(4):971-983.
2. Knight H, Veale E, Warren GJ, Knight MR: **The *sfr6* mutation in *Arabidopsis* suppresses low-temperature induction of genes dependent on the CRT/DRE sequence motif.** *Plant Cell* 1999, **11**(5):875-886.

Additional file:

Table S2

Relative variation of transcript steady-state content in Col, after the induction of DNA dsb damage.

Monitoring (qRT-PCR) the first 48 hours (0 to 2880 minutes) after exposure to ionising radiation.

	Amplicon	Minutes after exposure to radiation												
		**0**	**5**	**15**	**30**	**45**	**60**	**120**	**240**	**360**	**480**	**600**	**1440**	**2880**
**Fold Var**	**51**	1	186.6702	149.6269	168.1661	272.4258	284.1161	316.9157	204.8426	125.2894	121.9149	78.40245	29.25609	15.06317
	**1**	1	53.54165	40.92687	46.83357	67.7998	87.59845	97.88312	79.26297	48.20304	49.13953	28.89843	14.08289	6.599833
	**z**	1	0.583032	0.477297	0.397605	0.599464	0.651103	0.726668	0.832086	0.509164	0.325115	0.256745	0.347744	1.65335
	**3**	1	1.181406	0.818527	0.914004	1.301154	1.534173	1.709063	2.143187	2.012264	2.285025	1.641977	1.27767	1.347655
	**1m**	1	63.29162	47.03733	54.0387	89.11386	99.8299	117.6006	86.43222	56.60425	58.06457	32.71513	15.70655	7.500081
	**at**	1	0.723522	0.563913	0.481735	0.855646	0.754292	0.602345	1.158838	1.501672	1.572517	1.241766	0.938196	1.198238
**St Dev**	**51**	0.175723	35.4456	32.8832	15.00624	33.49177	46.18903	16.95891	48.43606	23.19096	20.60143	13.95699	5.240566	6.76956
	**1**	0.309898	6.755377	3.982508	3.016104	7.519523	11.0052	12.14928	15.6882	4.244197	5.012432	1.878921	1.910037	2.18174
	**z**	0.223832	0.080524	0.064414	0.135279	0.095736	0.192384	0.271053	0.456249	0.115869	0.08864	0.117311	0.08719	0.916944
	**3**	0.201424	0.141439	0.0454	0.057288	0.069831	0.252478	0.092328	0.489181	0.178863	0.240239	0.136999	0.150864	0.140895
	**1m**	0.224794	6.230258	5.435949	11.35453	18.06941	12.45908	25.78666	5.992909	11.67872	13.67503	5.84014	2.166994	3.066805
	**at**	0.313456	0.087211	0.081467	0.073366	0.162164	0.047643	0.092462	0.371092	0.14435	0.237287	0.090461	0.072242	0.168233

--- **amplicon**

AtRAD21.1 qRT-PCR products amplified with two different primer pairs ……... .. . (**1m** and **1**)
AtRAD21.2 qRT-PCR product amplified with one primer pair .…………..…….. ... (**z**)
AtRAD21.3 qRT-PCR product amplified with one primer pair .…………..…….. ... (**3**)

Controls:

- positive control: ***AtRAD51*** qRT-PCR product amplified with one primer pair ………….. (**51**)
- control non-responsive to radiation induced expression: ***AtATM*** qRT-PCR product amplified with one primer pair ……………………………………………………………….. (**at**)
- reference control: ***Actin2*** qRT-PCR product amplified with one primer pair …………...… (**ac**)
- reference control: ***AtEF1 αA4*** qRT-PCR product amplified with one primer pair ……..… (**f1**)

--- **minutes after the end of irradiation**

Samples harvested at **0** minutes after the end of the exposure to ionising radiation (non-irradiated) ………… **(0)**
Samples harvested **5** minutes after the end of the exposure to ionising radiation (316 Gy; 2.65 Gy/minute) …… **(5)**
Samples harvested **15** minutes after the end of the exposure to ionising radiation (316 Gy; 2.65 Gy/minute) …. **(15)**
Samples harvested **30** minutes after the end of the exposure to ionising radiation (316 Gy; 2.65 Gy/minute) …. **(30)**
Samples harvested **45** minutes after the end of the exposure to ionising radiation (316 Gy; 2.65 Gy/minute) …. **(45)**
Samples harvested **1** hour after the end of the exposure to ionising radiation (316 Gy; 2.65 Gy/minute) ……… **(60)**
Samples harvested **2** hours after the end of the exposure to ionising radiation (316 Gy; 2.65 Gy/minute) ….… **(120)**
Samples harvested **4** hours after the end of the exposure to ionising radiation (316 Gy; 2.65 Gy/minute) ….… **(240)**
Samples harvested **6** hours after the end of the exposure to ionising radiation (316 Gy; 2.65 Gy/minute) ….… **(360)**
Samples harvested **8** hours after the end of the exposure to ionising radiation (316 Gy; 2.65 Gy/minute) ….… **(480)**
Samples harvested **10** hours after the end of the exposure to ionising radiation (316 Gy; 2.65 Gy/minute) …... **(600)**
Samples harvested **24** hours after the end of the exposure to ionising radiation (316 Gy; 2.65 Gy/minute) ….. **(1440)**
Samples harvested **48** hours after the end of the exposure to ionising radiation (316 Gy; 2.65 Gy/minute) ..… **(2880)**

The sequences of primer pairs amplifying the qRT-PCR amplicons (**1m**, **1**, **z**, **3**, **51 at**) are provided in Additional file Table S3.

Fold Var - Relative variation of transcript content

St Dev - Standard deviation

Col - wild-type Columbia-0

Non-irradiated samples (0 minutes) were used as the reference value (with the arbitrary value: 1). Irradiated samples were exposure to ionising radiation: 316 Gy; 2.65 Gy/minute; source: Co60.

Additional file:

Table S3

Primers for qRT-PCR quantification of *AtRAD21* transcript steady state levels variation after exposure to ionising radiation.

Gene	primer	Primer sequence	PCR product; amplicon length (and name)	Efficiency (%) and R^2
AtRAD21.1 <u>*At5g40840*</u>	qRTmR21-1L	CTTTCATCTGCATTCACCTCATC	87 bp (**1m**)	99.1% R^2 = 0.986
	qRTmR21-1R	CTTTGAGGAGCACCCGTCTG		
AtRAD21.1 <u>*At5g40840*</u>	qRTR21-1L	GAACAGAGGGAAAGAGAAGAAGAAG	110 bp (**1**)	91.9% R^2 = 0.993
	qRTR21-1R	GTCTTCAACACCAAAGTCTCGTAG		
AtRAD21.2 <u>*At3g59550*</u>	qRTR21-2Lz	CTGGTGCTTCAGCCATCTCCTC	108 bp (**z**)	94.8% R^2 = 0.994
	qRTR21-2Rz	AAGCCTTTCACTTATGTTCTTGTTG		
AtRAD21.3 <u>*At5g16270*</u>	qRTR21-3Lx	CACATGACACAGGATTTTTGAACG	132 bp (**3**)	100% R^2 = 0.989
	qRTR21-3Rx	CCACAGCCCTAGTACGAGAAGAC		
AtRAD51 <u>*At5g20850*</u>	qRTR51-L	TCGAACAGCTTCAGGCAGCAG	102 bp (**51**)	102.2% R^2 = 0.998
	qRTR51-R	CCTTCCTCGGAGTATAAGCAACAC		
AtATM <u>*At3g48190*</u>	rtATM-L	GAATGGCAGCGTCCATAACT	95 bp (**at**)	105.8% R^2 = 0.998
	rtATM-R	TGCACCACAAACTACTGCTGA		
Actin2 <u>*At3g18780*</u>	qRTAC2-L	GAGAGAAAGTAAGAGATAATCCAGGAG	101 bp	94% R^2 = 0.998
	qRTAC2-R	GCCATTTTTTATGAGCTTGGAAAG		
AtEF1αA4 <u>*At5g60390.1* and *.2*</u>	qRTEF1-Lx	CTTACTTGCAGCTATGGGTAAAGAGA	111 bp	103.2% R^2 = 0.998
	qRTEF1-Rx	CCACCAAGCTTATAGATCAAGTGAC		

All primers were specifically designed for this study.

Additional file:

Table S4 **Number of true leaves per seedling**

15 days after irradiation

0 Gy - 1st assay

		Number of seedlings with 0, 1, 2 , 3, 4, 5, 6 and 7 true leaves							
Total number of seedlings	Genotype	**0**	**1**	**2**	**3**	**4**	**5**	**6**	**7**
19	**Col**	0	0	2	6	7	3	1	0
53	***atrad21.1***	5	0	5	9	17	13	4	0
38	***atrad21.3***	0	4	9	6	15	4	0	0
27	***atrad21.1 atrad21.3***	4	0	3	4	10	4	1	1
26	**Ws**	1	0	3	6	12	4	0	0
35	***atku80***	0	0	2	10	18	4	0	1
55	***atku80 atrad21.1***	0	1	6	7	21	18	2	0

0 Gy - 2nd assay

		Number of seedlings with 0, 1, 2 , 3, 4, 5, 6 and 7 true leaves							
Total number of seedlings	Genotype	**0**	**1**	**2**	**3**	**4**	**5**	**6**	**7**
60	**Col**	2	0	9	9	32	7	1	0
34	***atrad21.1***	0	0	3	4	10	14	3	0
56	***atrad21.3***	7	4	8	9	9	12	6	1
45	***atrad21.1 atrad21.3***	0	0	8	6	8	8	11	4
29	**Ws**	0	0	5	7	10	7	0	0
21	***atku80***	0	0	0	5	10	6	0	0
43	***atku80 atrad21.1***	0	0	6	8	15	14	0	0

0 Gy - Compiled data from both assays

		Number of seedlings with 0, 1, 2 , 3, 4, 5, 6 and 7 true leaves							
Total number of seedlings	Genotype	**0**	**1**	**2**	**3**	**4**	**5**	**6**	**7**
79	**Col**	2	0	11	15	39	10	2	0
87	***atrad21.1***	5	0	8	13	27	27	7	0
94	***atrad21.3***	7	8	17	15	24	16	6	1
72	***atrad21.1 atrad21.3***	4	0	11	10	18	12	12	5
55	**Ws**	1	0	8	13	22	11	0	0
56	***atku80***	0	0	2	15	28	10	0	1
98	***atku80 atrad21.1***	0	1	12	15	36	32	2	0

15 days after irradiation

100 Gy - 1st assay

		Number of seedlings with 0, 1, 2 , 3, 4, 5, 6 and 7 true leaves							
Total number of seedlings	genotype	**0**	**1**	**2**	**3**	**4**	**5**	**6**	**7**
39	**Col**	4	3	3	7	10	6	5	1
58	***atrad21.1***	15	12	10	9	5	7	0	0
55	***atrad21.3***	10	6	16	7	10	2	4	0
36	***atrad21.1 atrad21.3***	20	10	5	1	0	0	0	0
54	**Ws**	2	4	15	22	9	1	1	0
161	***atku80***	134	26	1	0	0	0	0	0
165	***atku80 atrad21.1***	150	13	2	0	0	0	0	0

100 Gy - 2nd assay

		Number of seedlings with 0, 1, 2 , 3, 4, 5, 6 and 7 true leaves							
Total number of seedlings	genotype	**0**	**1**	**2**	**3**	**4**	**5**	**6**	**7**
24	**Col**	1	5	6	2	7	2	1	0
29	***atrad21.1***	1	2	9	2	9	5	1	0
46	***atrad21.3***	0	8	8	11	12	5	2	0
47	***atrad21.1 atrad21.3***	23	13	5	3	2	1	0	0
32	**Ws**	1	0	3	14	13	1	0	0
129	***atku80***	117	12	0	0	0	0	0	0
154	***atku80 atrad21.1***	151	3	0	0	0	0	0	0

100 Gy - Compiled data from both assays

		Number of seedlings with 0, 1, 2 , 3, 4, 5, 6 and 7 true leaves							
Total number of seedlings	genotype	**0**	**1**	**2**	**3**	**4**	**5**	**6**	**7**
63	**Col**	5	8	9	9	17	8	6	1
87	***atrad21.1***	16	14	19	11	14	12	1	0
101	***atrad21.3***	10	14	24	18	22	7	6	0
83	***atrad21.1 atrad21.3***	43	23	10	4	2	1	0	0
86	**Ws**	3	4	18	36	22	2	1	0
290	***atku80***	251	38	1	0	0	0	0	0
319	***atku80 atrad21.1***	301	16	2	0	0	0	0	0

15 days after irradiation

150 Gy - 1st assay

		Number of seedlings with 0, 1, 2 , 3, 4, 5, 6 and 7 true leaves							
Total number of seedlings	genotype	0	1	2	3	4	5	6	7
61	**Col**	17	13	6	10	11	3	1	0
72	***atrad21.1***	32	15	7	12	6	0	0	0
68	***atrad21.3***	28	23	6	7	4	0	0	0
6	***atrad21.1 atrad21.3***	5	1	0	0	0	0	0	0
68	**Ws**	18	26	14	9	0	0	1	0
204	***atku80***	203	1	0	0	0	0	0	0
283	***atku80 atrad21.1***	271	5	6	1	0	0	0	0

150 Gy - 2nd assay

		Number of seedlings with 0, 1, 2 , 3, 4, 5, 6 and 7 true leaves							
Total number of seedlings	genotype	**0**	**1**	**2**	**3**	**4**	**5**	**6**	**7**
63	**Col**	6	6	12	15	14	10	0	0
86	***atrad21.1***	47	18	12	3	3	1	2	0
49	***atrad21.3***	11	15	7	7	5	3	1	0
106	***atrad21.1 atrad21.3***	62	20	15	7	2	0	0	0
62	**Ws**	13	26	15	7	1	0	0	0
103	***atku80***	103	0	0	0	0	0	0	0
138	***atku80 atrad21.1***	136	2	0	0	0	0	0	0

150 Gy - Compiled data from both assays

		Number of seedlings with 0, 1, 2 , 3, 4, 5, 6 and 7 true leaves							
Total number of seedlings	genotype	**0**	**1**	**2**	**3**	**4**	**5**	**6**	**7**
124	**Col**	23	19	18	25	25	13	1	0
158	***atrad21.1***	79	33	19	15	9	1	2	0
117	***atrad21.3***	39	38	13	14	9	3	1	0
112	***atrad21.1 atrad21.3***	67	21	15	7	2	0	0	0
130	**Ws**	31	52	29	16	1	0	1	0
307	***atku80***	306	1	0	0	0	0	0	0
421	***atku80 atrad21.1***	407	7	6	1	0	0	0	0

Table S4 legend:

Number of emerging and fully expanded true leaves in seedlings (Col, Ws, *atrad2.1, atrad21.3, atrad21.1 atrad21.3, atku80, atku80 atrad21.1*) non-exposed to ionising radiation (0 Gy) and exposed to 100 Gy and 150 Gy of ionising radiation (γ-rays; 3.25 Gy/minute; source: Cs137).

Tables show the number of seedlings with 0, 1, 2, 3, 4, 5, 6 or 7 true leaves observed 15 days after irradiation (15 DAI) of pre-imbibed seed, in two independent biological replicas (assays). Col- Wild type Col-0. Ws- Wild type Wassilewskija-1

Additional file:

Table S5 Mean, Mode and Median (true leaves per seedling)

15 days after irradiation

0 Gy

	**Mean**	σ up (xi ≥ mean)	σ down (xi < mean)	**Mode**	**Median**	σ up (xi ≥median)	σ down (xi<median)
Col	**3.608**	0.84992	1.49065	**4**	**4**	0.67505	1.53741
atrad21.1	**3.908**	1.01671	2.156	**4, 5**	**4**	1.12444	1.71499
atrad21.3	**3.255**	1.60607	1.76092	**4**	**3.5**	1.39719	1.96574
atrad21.1 atrad21.3	**4.042**	1.86437	1.70486	**4**	**4**	1.67083	1.83615
Ws	**3.6**	0.87178	1.34731	**4**	**4**	0.63246	1.5172
atku80	**3.893**	0.75504	1.09327	**4**	**4**	0.82375	0.92296
atku80 atrad21.1	**3.939**	0.79884	1.57483	**4**	**4**	0.90351	1.22474

100 Gy

	**Mean**	σ up (xi ≥ mean)	σ down (xi < mean)	**Mode**	**Median**	σ up (xi ≥median)	σ down (xi<median)
Col	**3.238**	1.71959	1.88684	**4**	**4**	1.14087	2.54146
atrad21.1	**2.379**	1.89554	1.58079	**2**	**2**	2.09542	1.35473
atrad21.3	**2.723**	1.6137	1.65172	**2**	**3**	1.43506	1.8532
atrad21.1 atrad21.3	**0.819**	1.33629	0.82897	**0**	**0**	1.93882	0
Ws	**2.930**	0.84294	1.53077	**3**	**3**	0.95235	1.20515
atku80	**0.138**	0.90167	0.13821	**0**	**0**	0.5382	0
atku80 atrad21.1	**0.0627**	1.0945	0.0628	**0**	**0**	0.3879	0

150 Gy

	**Mean**	σ up (xi ≥ mean)	σ down (xi < mean)	**Mode**	**Median**	σ up (xi ≥median)	σ down (xi<median)
Col	**2.427**	1.62414	1.73462	**3, 4**	**3**	1.17775	2.22136
atrad21.1	**1.069**	2.15533	0.90316	**0**	**0.5**	2.06155	0.50319
atrad21.3	**1.393**	2.01461	1.03599	**0**	**1**	1.95243	0.82357
atrad21.1 atrad21.3	**0.714**	1.37173	0.71968	**0**	**0**	1.77281	0
Ws	**1.292**	1.38899	0.82798	**1**	**1**	1.3978	0.69597
atku80	**0.004**	0.99674	0.00326	**0**	**0**	0.08071	0
atku80 atrad21.1	**0.0523**	1.64184	0.05232	**0**	**0**	0.43592	0

Table S5 legend:

Mean, Mode and Median of the number of emerging and fully expanded true leaves in seedlings (Col, Ws, *atrad2.1, atrad21.3, atrad21.1 atrad21.3, atku80, atku80 atrad21.1*) non-exposed to ionising radiation (0 Gy) and exposed to 100 Gy and 150 Gy of ionising radiation.

Due to the detection of skewed data distributions at 100 Gy and 150 Gy, the values of the Median and Mode are shown to illustrate better the higher incidence of seedlings with 0 true leaves, 15 days after the irradiation, in the genotypes that are more hypersensitive to ionising radiation exposure. The *atrad21.1 atrad21.3* and the *atku80 atrad21.1* double mutants and the *atku80* single mutant have a particularly high incidence of seedlings with none or few true leaves at 100 Gy, and also at 150 Gy, illustrating their clear hypersensitivity to ionising radiation exposure.

Mean, Median and Mode obtained from the combined data (Additional file Table S4) from both independent biological replicas (assays) combined. σ up - standard deviation of data with equal or higher value than the mean (or median); σ down - standard deviation of data with lower value than the mean (or median). 0 Gy: non-exposed to ionising radiation. 100 Gy, 150 Gy: exposed to 100 Gy (or 150 Gy) of ionising radiation (γ-rays; 3.25 Gy/minute; source: Cs137). Col- Wild type Col-0. Ws- Wild type Wassilewskija-1

Additional file:

Table S6

Comet assay data

DNA dsb repair during the first 3 hours (0 to 180 minutes (tx)) after DNA dsb induction with 30 µg/ml Bleomycin

(in Col, *atrad21.1*, *atrad21.3* and *atrad21.1 atrad21.3* mutants).

Amount of DNA in comet tail (**% Tail DNA**) decreases as a result of DNA dsb damage repair.
Kinetics (K) of DNA dsb damage repair

	% Tail DNA	SE	C(tx)	K(tx)	tx
**1 -** *atrad21.1*	65.5	4.0	38.6	100.0	0
	63.4	8.7	36.5	94.6	3
	59.0	8.5	32.2	83.2	5
	50.8	5.2	24.0	62.1	10
	48.2	7.7	21.4	55.4	20
	35.4	2.4	8.5	22.1	60
	28.7	1.6	1.8	4.7	180
	26.8	1.3			control
**3 -** *atrad21.3*	64.8	4.8	39.0	100.0	0
	64.3	9.9	38.6	98.9	3
	56.0	7.8	30.3	77.5	5
	53.9	8.8	28.2	72.2	10
	49.5	8.0	23.8	60.9	20
	33.7	3.0	7.9	20.3	60
	29.3	1.7	3.5	9.0	180
	25.7	1.8			control
**D -** *atrad21.1 atrad21.3*	71.5	7.2	45.6	100.0	0
	69.8	5.2	43.9	96.4	3
	55.1	4.2	29.3	64.2	5
	43.4	2.1	17.5	38.4	10
	40.1	3.3	14.3	31.3	20
	32.0	1.5	6.1	13.3	60
	28.1	1.4	2.2	4.8	180
	25.9	1.6			control
**C -** Col	72.3	6.3	52.9	100.0	0
	65.2	7.1	45.8	86.6	3
	55.9	7.3	36.5	68.9	5
	40.6	3.0	21.2	40.2	10
	36.0	2.3	16.6	31.3	20
	28.5	1.1	9.1	17.1	60
	26.3	1.1	6.9	13.0	180
	19.4	1.2			control

Table S6 legend:

dsb - double strand break

% Tail DNA - percentage of total nuclear that has dsb fragments (tail of the comet)

SE - standard error

C(tx) - Increment of the amount of DNA dsb fragments (DNA in tail) in comparison to samples not exposed to DNA induction

C(tx) = % Tail DNA (tx) - % Tail DNA (control)

K(tx) - DNA dsb repair kinetics

K(tx) = C(tx) / C(t0) x 100

tx - time (minutes) after the DNA dsb induction treatment

t0 - 0 minutes (minutes) after the DNA dsb induction treatment

control - samples not treated with Bleomycin

Genotype:

1 - *atrad21.1* homozygous mutant

3 - *atrad21.3* homozygous mutant

D - *atrad21.1 atrad21.3* double homozygous mutant

C - Col ; wild-type Columbia-0

Additional file:

Table S7

Primers for mutants and complementation lines genotyping

Gene / mutant / construct	Primer name	PCR product	Primer sequence	Observations
atrad21.1 Salk_044851	LBa1 [1]	amplifies **mutant** gDNA only	TGGTTCACGTAGTGGGCCATCG	Annealing: 55°C, 45'' extention: 72°C, 1.5'
	51L [2]		GAGATGGTCACACAGAGAATTTAG	
AtRAD21.1 <u>*At5g40840*</u>	51L [2]	amplifies **wild type** gDNA only	see above	Annealing: 53°C, 45'' extention: 72°C, 1.5'
	51R [2]		CTCCTCTCAGGACAGTCAGTATG	
atrad21.3 Salk_076116	LBa1 [1]	amplifies **mutant** gDNA only	see above	Annealing: 58°C, 30'' extention: 72°C, 1.5'
	16L [2]		CTGTGTCATGTGCATTTTCCATGG	
atrad21.3 Salk_076116	LBa1 [1]	amplifies **mutant** gDNA only	see above	Annealing: 58°C, 30'' extention: 72°C, 1.5'
	16R [2]		CCGTGTAGAGGATTTACAGGTTG	
AtRAD21.3 <u>*At5g16270*</u>	16L [2]	amplifies **wild type** gDNA only	see above	Annealing: 58°C, 30'' extention- 72°C, 1.5'
	16R [2]		see above	
atku80 West *et al.*, 2002	LB Fel [3]	amplifies **mutant** gDNA only	GATTCTTTTTATGCATAGATGCAC	Annealing: 56°C, 45'' extention: 72°C, 2'
	K6 [3]		CTCCAAGACGCAGCCTTTAC	
atku80 West *et al.*, 2002	RB Fel +	amplifies **mutant** gDNA only	TCCCGCCTTCGGTTCCACC	Annealing: 56°C, 45'' extention: 72°C, 2'
	KR +		CGTATCTGCTATTGCAAGAGA	

AtKU80 <u>*At1g48050*</u>	K6 [3]	amplifies **wild type** gDNA only	see above	Annealing: 56°C, 45'' extention: 72°C, 2'
	KR +		see above	
Comp	GFPOUT +	amplifies **Comp** only	GTATGTTGCATCACCTTCAC	Annealing: 56°C, 45'' extention: 72°C, 2.5'
	3HOM6 +		GTAACGTGGTTTCGGTTGAG	

\+ primers designed for this study

Comp - complementation line and complementation construct gDNA *pARAD21.1-AtRAD21.1-GFP*-6x*His*

1. Alonso JM, Stepanova AN, Leisse TJ, Kim CJ, Chen H, Shinn P, Stevenson DK, Zimmerman J, Barajas P, Cheuk R, Gadrinab C, Heller C, Jeske A, Koesema E, Meyers CC, Parker H, Prednis L, Ansari Y, Choy N, Deen H, Geralt M, Hazari N, Hom E, Karnes M, Mulholland C, Ndubaku R, Schmidt I, Guzman P, Aguilar-Henonin L, Schmid M *et al.*: **Genome-wide insertional mutagenesis of *Arabidopsis thaliana*.** *Science* 2003, **301**(5633):653-657.
2. da Costa-Nunes JA, Bhatt AM, O'Shea S, West CE, Bray CM, Grossniklaus U, Dickinson HG: **Characterization of the three *Arabidopsis thaliana RAD21* cohesins reveals differential responses to ionizing radiation.** *J Exp Bot* 2006, **57**(4):971-983.
3. West CE, Waterworth WM, Story GW, Sunderland PA, Jiang Q, Bray CM: **Disruption of the Arabidopsis *AtKu80* gene demonstrates an essential role for AtKu80 protein in efficient repair of DNA double-strand breaks *in vivo*.** *Plant J* 2002, **31**(4):517-528.
